# Long‐term data reveal widespread phenological change across major US estuarine food webs

**DOI:** 10.1111/ele.14441

**Published:** 2024-12-31

**Authors:** Robert J. Fournier, Denise D. Colombano, Robert J. Latour, Stephanie M. Carlson, Albert Ruhi

**Affiliations:** ^1^ Department of Environmental Science, Policy, and Management University of California Berkeley Berkeley California USA; ^2^ Delta Science Program Delta Stewardship Council Sacramento California USA; ^3^ Virginia Institute of Marine Science William & Mary Gloucester Point Virginia USA

**Keywords:** climate change, estuaries, long‐term data, match–mismatch hypothesis, phenology

## Abstract

Climate change is shifting the timing of organismal life‐history events. Although consequential food‐web mismatches can emerge if predators and prey shift at different rates, research on phenological shifts has traditionally focused on single trophic levels. Here, we analysed >2000 long‐term, monthly time series of phytoplankton, zooplankton, and fish abundance or biomass for the San Francisco, Chesapeake, and Massachusetts bays. Phenological shifts occurred in over a quarter (28%) of the combined series across all three estuaries. However, phenological trends for many taxa (ca. 29–68%) did not track the changing environment. While planktonic taxa largely advanced their phenologies, fishes displayed broad patterns of both advanced and delayed timing of peak abundance. Overall, these divergent patterns illustrate the potential for climate‐driven trophic mismatches. Our results suggest that even if signatures of global climate change differ locally, widespread phenological change has the potential to disrupt estuarine food webs.

## Graphical abstract

Climate change is shifting the timing of organismal life‐history events. Although consequential food‐web mismatches can emerge if predators and prey shift at different rates, research on phenological shifts has traditionally focused on single trophic levels. Here, we analysed >2000 long‐term, monthly time series of phytoplankton, zooplankton, and fish abundance or biomass for the San Francisco, Chesapeake, and Massachusetts bays. Phenological shifts occurred in over a quarter (27%) of the combined series across all three estuaries. Additionally, divergent patterns between predator and prey illustrate the potential for climate‐driven trophic mismatch. Our results suggest that even if signatures of global climate change differ locally, widespread phenological change has the potential to disrupt estuarine food webs.

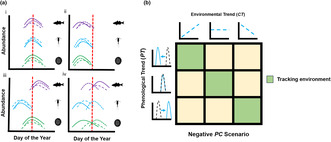

## PEER REVIEW

The peer review history for this article is available at https://www.webofscience.com/api/gateway/wos/peer‐review/10.1111/ele.14441.

## INTRODUCTION

Shifts in phenology, or the timing of events within an organism's life cycle, are a common response to global climate change. Phenological shifts have been observed in plants, invertebrates, and vertebrates across diverse systems—from mountain forests to marine fishes (Tang et al., 2016). Fluctuations in climatic conditions often drive variation in timing of migration (Goertler et al., 2021; Gordo, 2007) or peak abundance (Brown, 2016; Cohen et al., 2018). Indeed, ample research has examined the unique roles of temperature, precipitation, nutrient availability, or salinity as drivers of phenological change (Guinder et al., 2017; Kristiansen et al., 2016; McQueen & Marshall, 2017). While the occurrence and magnitude of phenological shifts appears to be increasing (Vitasse et al., 2022), few studies have leveraged long‐term data across taxonomic groups, hindering examination of phenological change within food webs (but see Thackeray et al., 2016, Cohen et al., 2018, Asch et al., 2019).

Phenological shifts can lead to asynchrony, or mismatches, between an organism and its environment. For instance, migratory organisms might arrive in breeding or rearing habitats when abiotic conditions are unsuitable (Satterthwaite et al., 2014), or when food resources are scarce (Post & Forchhammer, 2008). While phenological plasticity can promote persistence in fluctuating environments (Ovaskainen et al., 2013), population performance often depends on maintaining synchrony with interacting populations (Visser & Gienapp, 2019). Importantly, the rate or direction of phenological shifts might differ among interacting taxa—especially if climatic sensitivities vary (Chmura et al., 2019). If historically synchronous, interacting populations become decoupled, cascading impacts on community structure and functioning may follow (Thakur, 2020; Varpe & Fiksen, 2010). For example, phenological shifts have been shown to disrupt predator/prey interactions in frogs and dragonflies (Rasmussen & Rudolf, 2016), as well as mutualistic relationships between plants and pollinators (Rafferty et al., 2015). Understanding how species' climate sensitivities influence the potential for trophic mismatches is an active area of research (Leathers et al., 2024; Thomas et al., 2017), and one that requires quantifying long‐term environmental and biological trends both within and across trophic levels.

**FIGURE 1:**
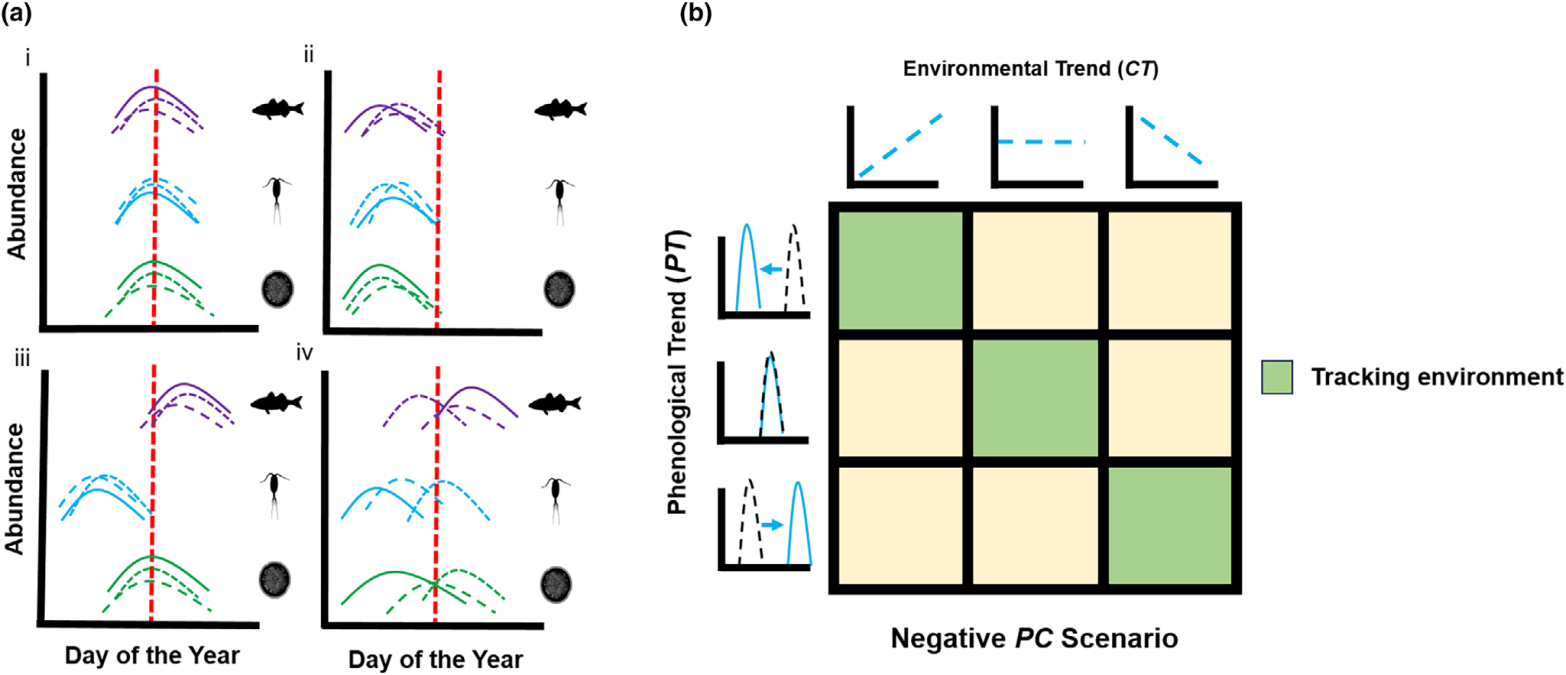
Potential phenological response types and relationships between phenological and environmental trends. (a) Conceptual diagram showing shifting phenologies within an estuarine community, with example population abundance curves for fishes (purple), zooplankton (blue), and phytoplankton (green). We display four potential scenarios where timing of peak abundance (represented as the calendar date of the peak) might shift relative to the historical date of peak abundance (red dashed line). In (i), there is no phenological change; in (ii) there is a uniform shift between trophic levels; in (iii), phenological shifts are consistent within, but not between trophic levels; and finally in (iv), phenological shifts diverge both within and between trophic levels. (b) Conceptual diagram showing relationships between the triplet of slopes produced by our model. The environmental trend slope (*CT*) shows whether the environmental covariate (temperature or salinity) is increasing through time (left column), remaining constant (middle), or decreasing (right). The phenological trend (*PT*) shows if the date of peak abundance is occurring earlier (top row), staying constant (middle), or delaying to later in the year (bottom). The final slope is the relationship between phenology and the environmental covariate. A positive slope indicates that an increase in the covariate would cause a delay in phenology (and vice versa) while a negative slope would indicate that an increase in the covariate value would cause an earlier peak. A non‐significant *PC* slope indicates no phenology/covariate relationship. Organisms are considered to be ‘tracking their environment’ if the combination of these three slopes is congruous. In this figure, we show the relationships assumed with a negative *PC* slope. Under this scenario, for example, an organism with an advancing phenology that exists in a warming climate is tracking temperature trends. With a positive *PC* slope, the combination of climatic and phenological scenarios in the opposite diagonal would be considered ‘tracking’.

At the community scale, phenological shifts may lead to a trophic mismatch depending on the magnitude or direction of change across and within trophic levels (see Figure 1a for hypothesized response types). Phenology may remain stable even if environmental conditions change (Figure 1a, i), or it may shift in a consistent way across trophic levels, allowing the food web to remain coupled (Figure 1a, ii). Shifts may also differ in magnitude or direction among trophic levels (Figure 1a, iii), increasing trophic mismatch potential if the diversity of responses within trophic levels is low. However, a high diversity of species‐specific phenological trends within trophic levels (i.e., response diversity) would decrease trophic mismatch potential even when phenological shifts are widespread (Figure 1a, iv). Importantly, congruency between phenological and climatic trends might be indicative of climate forcing (Chmura et al., 2019), while divergence could suggest an inability to track the direction and/or rate of environmental change and thus the potential accrual of ‘climatic debt’ (Duchenne et al., 2021, Figure 1b).

Estuaries are particularly vulnerable to climate change due to their transitional, variable nature (Lauchlan & Nagelkerken, 2020), and many taxa use them as breeding or nursery grounds (Beck et al., 2001; Colombano et al., 2020), as refuge from predation (Simenstad et al., 1982), or as migration corridors (Koeller et al., 2009; Otero et al., 2014). In estuarine food webs, temperature fluctuations govern many aspects of community structure, including growth rates, migration timing, and planktonic blooms (Apple et al., 2006; Incze et al., 1980; Kromkamp & Engeland, 2010). Additionally, salinity levels influence habitat use and life cycle cues (Harrison & Whitfield, 2006; Lane et al., 2007). Despite the natural dynamism of estuarine systems, many taxa have developed population cycles that maintain historical synchrony between interacting species (Marques et al., 2006). However, estuaries globally are experiencing climate‐induced changes in temperature (Scanes et al., 2020) and salinity (Ghalambor et al., 2021). Because these variables control fish and plankton population dynamics, climatic fluctuations could drive phenological shifts that disrupt estuarine food webs by pushing population cycles outside of historically synchronous windows (Asch et al., 2019; Chevillot et al., 2017; Langan et al., 2021). Adding to this complexity, the same suite of global change drivers can lead to divergent climatic trends at local scales (Zhang et al., 2022). Thus, understanding how local, climate‐driven phenological shifts may create potential for trophic mismatches is particularly consequential for estuarine ecosystems.

Here, we explore phenological shifts, their climatic drivers, and the potential for trophic mismatches using long‐term monitoring data from three major North American estuaries: the San Francisco, Chesapeake, and Massachusetts bays. We hypothesized that: (1) *Phenological shifts would be common in all three systems, and shifts would predominantly involve an advancement of peak abundance dates*. Because many taxa peak in the spring, advancing phenologies might help ameliorate the negative effects of warming (Cohen et al., 2018). (2) *The climatic drivers of phenological shifts would vary between systems and among taxonomic groups*. For example, long‐term drought in the San Francisco Estuary might exacerbate the effects of salinity in that system when compared to Massachusetts and Chesapeake bays (Hutton et al., 2016). Furthermore, these varying sensitivities might drive diverse phenological trends within a given trophic level, suggesting that taxa within the same trophic guild can respond differently to the same stressor (Mori et al., 2013). Finally, (3) *Divergent phenological patterns between trophic levels might drive potential for trophic mismatches between fish predators and their potential prey*. For instance, taxa with smaller body size and shorter lifespans might respond more strongly to higher‐frequency climatic variation than longer‐lived taxa (Farnsworth et al., 1995; Woodward et al., 2005). Importantly, divergent phenological patterns might scale down to localized food webs with the potential to influence trophic interactions.

To assess these hypotheses, we fitted trivariate meta‐regression models that jointly estimate relationships between phenology, climatic variables (temperature and salinity), and time (following Cohen et al., 2018). This model structure facilitates examining phenological shifts within and across food webs while maintaining the context of locally changing climatic conditions.

## METHODS

### Data sources and filtering

We compiled and filtered long‐term data for fishes, zooplankton, phytoplankton, temperature, and salinity from the San Francisco, Chesapeake, Massachusetts bays. For the San Francisco Bay, we obtained fish and water quality data from the California Department of Fish and Wildlife's San Francisco Bay Study (CDFW, 2023a), and zooplankton and phytoplankton data from the Environmental Monitoring Program (CDFW, 2023b). For Chesapeake Bay, we obtained fish and water quality data from the Virginia Institute of Marine Science's Chesapeake Multispecies Monitoring and Assessment Program (Latour et al., 2023); and zooplankton, phytoplankton, and associated water quality from the Chesapeake Bay Program (CBP, 2023). For Massachusetts Bay, we obtained phytoplankton, zooplankton, and water quality data from the Massachusetts Water Resources Authority's Water Column Monitoring Program (MWRA, 2023). All data were monthly, spanned at least 10 years (range 10–49 years; mean time series length ca. 28 years), and were examined for quality. We selected a minimum length of 10 years to facilitate both comparison of disparate sampling schemes and to enhance detection of linear patterns in oscillating monthly data (e.g., Mann & Lees, 1996). See Supporting Information S1 for details on data collection, filtering, and quality control.

For each combination of taxa, sampling location, and estuary, we calculated three Pearson correlation coefficients, capturing: (1) the relationship between phenology (adjusted yearly date of peak abundance) and time (hereafter, *PT*); (2) the relationship between the climatic variables and time (hereafter, *CT*); and (3) the relationship between phenology and climate (hereafter, *PC*). For each taxa and site, the triplet of coefficients was used to calculate Fisher's Z transformed effect sizes that entered a meta‐regression model (see raw coefficients; Fournier et al., 2023). Because taxonomic groups might respond to environmental trends across different temporal scales, we compared the models that incorporate climatic trends at annual vs. seasonal scales. For the annual models, we calculated mean annual temperature and salinity for each taxa/sampling location/estuary stratum. For the seasonal models, we calculated trends associated with temperature and salinity at the timing of peak abundance for that taxa/sampling location. Although temperature and salinity can interact, here we examined these variables in isolation. We primarily report the results from our annual models in the main text to facilitate comparability among monitoring programs with different sampling frequencies (Cohen et al., 2018). The results from seasonal models are shown in Supporting Information S5.

### Trivariate mixed‐effects meta regression model

We used trivariate mixed‐effects meta regression models to jointly analyse relationships between phenology, time, and each climatic variable. These models account for the non‐independence of effect sizes that arises due to shared variables (e.g., phenology is part of both *PT* and *PC*; Viechtbauer, 2010). In matrix notation, the trivariate meta regression model follows the following equation:

z=Wβ+ε+u,

where *z* is a vector of all the Fisher transformed effect sizes. For each *i*th group of *m* number of taxa/location strata, there are effect sizes representing three relationships (*PT, PC, CT*). Thus, *z* has a length of k=m×3. *W* is a regression design matrix with $m\times \left(j+1\right)$ dimensions with *j* number of covariates. The first column of *W* represents the model intercept (if present) and contains only ones. The regression coefficients of the model are defined in *β* which is a vector of length $\left(j+1\right)\times 3$. The within‐population variance–covariance matrix, ε is a blocked structure (one 3 × 3 matrix for each stratum of effect sizes at each sampling location) wherein the main diagonal of each block represents the sampling variance of each of the Fisher transformed effect sizes following the equation:

$$\mathrm{var}\left(Z_{\mathrm{PT}}=\mathrm{var}\left(Z_{\mathrm{CT}}\right)=\mathrm{var}\left(Z_{\mathrm{PC}}\right)=\frac{1}{n-3}\right),$$

where $Z_{\mathrm{PT}}$ is the effect size of phenology versus time (*PT*), $Z_{\mathrm{CT}}$ is the effect size for climate versus time (*CT*), and $Z_{\mathrm{PC}}$ is the effect size of phenology versus climate (*PC*). ε also accounts for non‐independence of effect sizes that share common variables in the estimated sampling covariances in the matrix off‐diagonal. For each pair of effect sizes that share an independent variable, the covariance between two Fisher transformed effect sizes (e.g., cov($Z_{\mathrm{PT}}$, $Z_{\mathrm{PC}}$)) is calculated using the untransformed Pearson correlation coefficients (*p*), following:

$$\mathrm{cov}=\frac{p_{\mathrm{PC}}\left(1-p_{\mathrm{CT}}^{2}+.05\times p_{\mathrm{PT}}\times p_{\mathrm{CT}}\times p_{\mathrm{PC}}\right)-0.5\left(p_{\mathrm{PT}}\times p_{\mathrm{CT}}\right)\left(1-p_{\mathrm{PT}}^{2}+p_{\mathrm{CT}}^{2}\right)\;}{\left(n-3\right)\left(\left(1-p_{\mathrm{PT}}^{2}\right)\times \left(1-p_{\mathrm{CT}}^{2}\right)\right)}.$$

The main effects of the model, *u*, are assumed to have a multivariate normal (MVN) distribution wherein the between‐population variance, $\tau^{2}$, for each correlation triplet (*PT, CT, PC*) is represented along the diagonal of a blocked matrix, and the covariance estimates for all levels of the random effect variables are estimated in the off diagonals. In all models, we included random effects that control for variation in time series start and end dates, as well as a nested random effect that accounts for station identity effects. Maximum likelihood estimations were performed using the rma.mv function in the metafor R package (Viechtbauer, 2010). We fitted models at both the estuary‐wide and sub‐estuary level scales. Our sub‐estuary models represent more realistic food webs by examining taxa found within limited geographical regions that are more likely to interact through space and time. We did not include Chesapeake Bay zooplankton in regional food web models as sampling stopped in 2002. The primary output of these models is a triplet of linearized slopes representing three relationships: phenology vs time, phenology vs climate, and climate vs time. A slope was considered significant if the 95% confidence interval for the estimate did not include zero.

To further explore the diversity of phenological (*PT*) and climatic trends (*CT*) at each estuary, we plotted these slopes against each other, and fitted Bayesian 95% confidence ellipses around the data. We quantified the area of each ellipse as well as the amount of overlap among ellipses representing our three broad taxonomic groups (fishes, zooplankton, phytoplankton). All ellipse calculations were performed using the SIBER package in R (Jackson et al., 2019). We also assessed whether a given organism's phenology was tracking climatic conditions. To be considered tracking, an organism had to (1) be shifting its phenology (significant *PT* slope); (2) exist in a changing climate (significant *CT* slope); and (3) have a significant relationship between phenology and climate that is consistent with the direction of the *PT* and *CT* slopes. For example, an organism advancing its phenology (negative *PT* slope) in a warming climate (positive *CT* slope) must have a negative *PC* slope. Additionally, we considered organisms with a static phenology under a static climate to be tracking, as time would not decouple that organism from its climate envelope. Conversely, organisms that either (1) shifted phenologies under static climates; (2) did not shift phenologies under changing climates; or (3) had inconsistent relationships between phenologies and climatic conditions, were all considered to ‘not track’ climatic variables (Figure 1b). Finally, we examined system‐wide abundance trends to understand if the ability of an organism to track climatic conditions was associated with its population dynamics (e.g., declining population size due to an inability to track climatic trends).

We used these model outputs to assess if phenological and climatic patterns comported with our hypotheses. To assess *Hypothesis 1*, that phenological shifts would be occurring and that phenologies would be predominantly advancing, we examined the *PT* slopes. Significant *PT* slopes are indicative of phenological change, while the sign of the slope indicates directionality—negative slopes represent phenological advances, and positive slopes indicate phenological delays. For *Hypothesis 2*, that the climatic drivers of phenological change would differ among systems and taxonomic groups, we explored if phenological sensitivities varied by environmental covariate, estuary, and trophic level by fitting a three‐way ANOVA (Quinn & Keough, 2002) on estimated *PC* slopes. Additionally, we compared the rates of environmental tracking for each variable and estuary by conducting Chi‐squared tests. To explore *Hypothesis 3*, that divergent phenological patterns between trophic groups might increase the potential for trophic mismatches, we first compared the variance of phenological trends between groups by conducting a Levene's test for homogeneity of variances on *PT* slopes. Finally, we assessed the divergence of phenological patterns in more localized food webs by fitting ANOVAs on the *PT* slopes from our regional food web models.

## RESULTS

### Environmental and phenological trends

**FIGURE 2:**
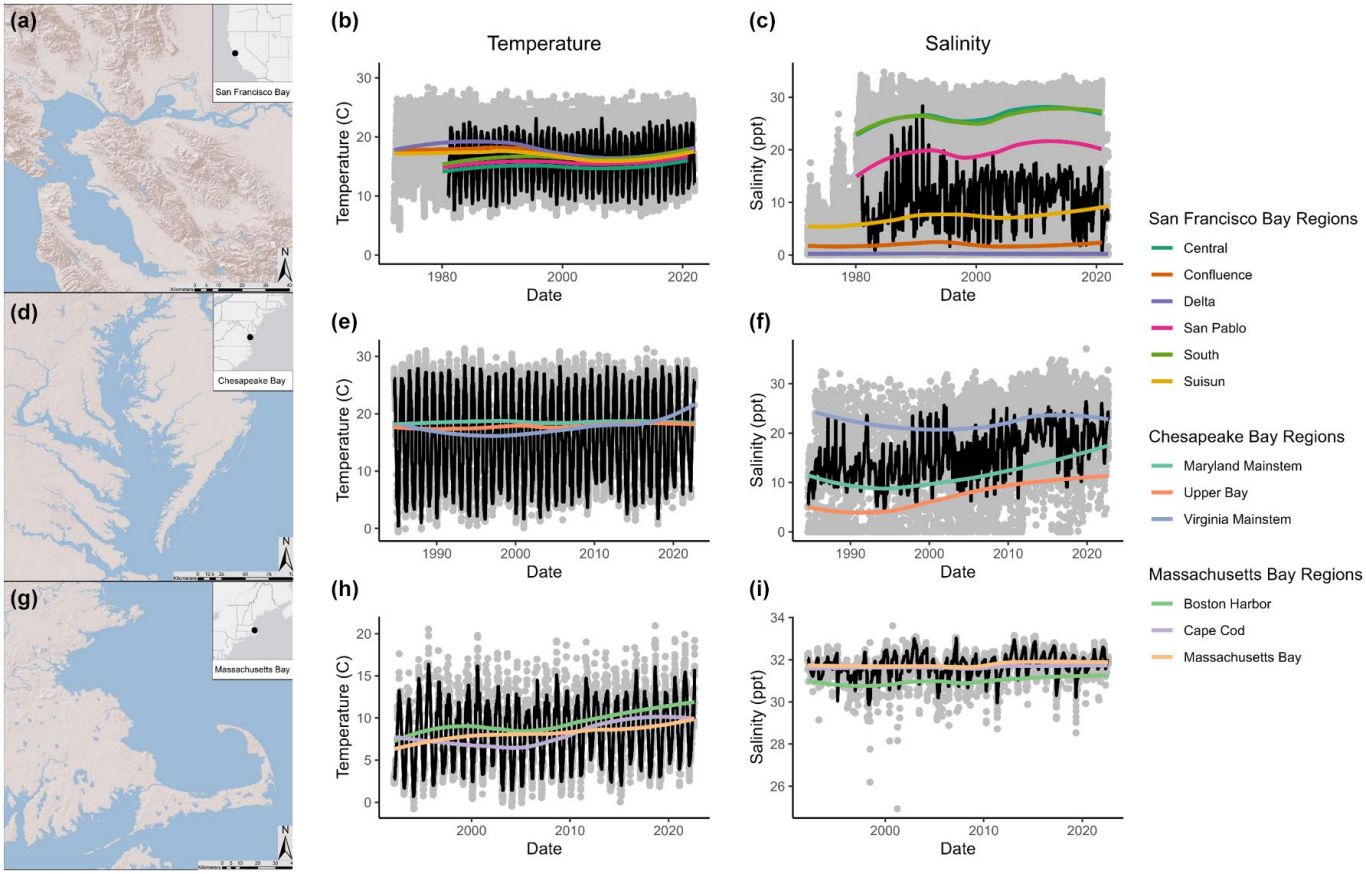
Study estuaries and associated long‐term environmental trends. Left column: maps detailing the location of the San Francisco Bay, Chesapeake Bay, and Massachusetts Bay. Central and right columns: long‐term climatic trends (temperature or salinity) across all recording stations of each estuary. Individual readings are shown as grey dots, and monthly temperature and salinity mean values (across stations recording data that month) are shown in black. LOESS trends at each of the regions within the estuary are shown in different colours. See distribution of environmental trends experienced by taxa at each estuary in Supplementary Figure S4.

In each estuary, both temperature and salinity tended to increase overall, but substantial spatial variation existed (Figure 2). Accordingly, taxa experienced a variety of local environmental trends, identified by significant *CT* slopes (33.3–64.2% warming, 0–15.15% cooling; 4.76–69.7% increasing salinity, 0–10.7% decreasing salinity; see Supporting Information S2). These trends were associated with widespread shifts in the timing of peak abundance, identified as significant *PT* slopes. Overall, 28% of the modelled taxa displayed significant phenological shifts, and shifting taxa disproportionately advanced their phenology (temperature: 85% advancing; salinity: 72% advancing).

**FIGURE 3:**
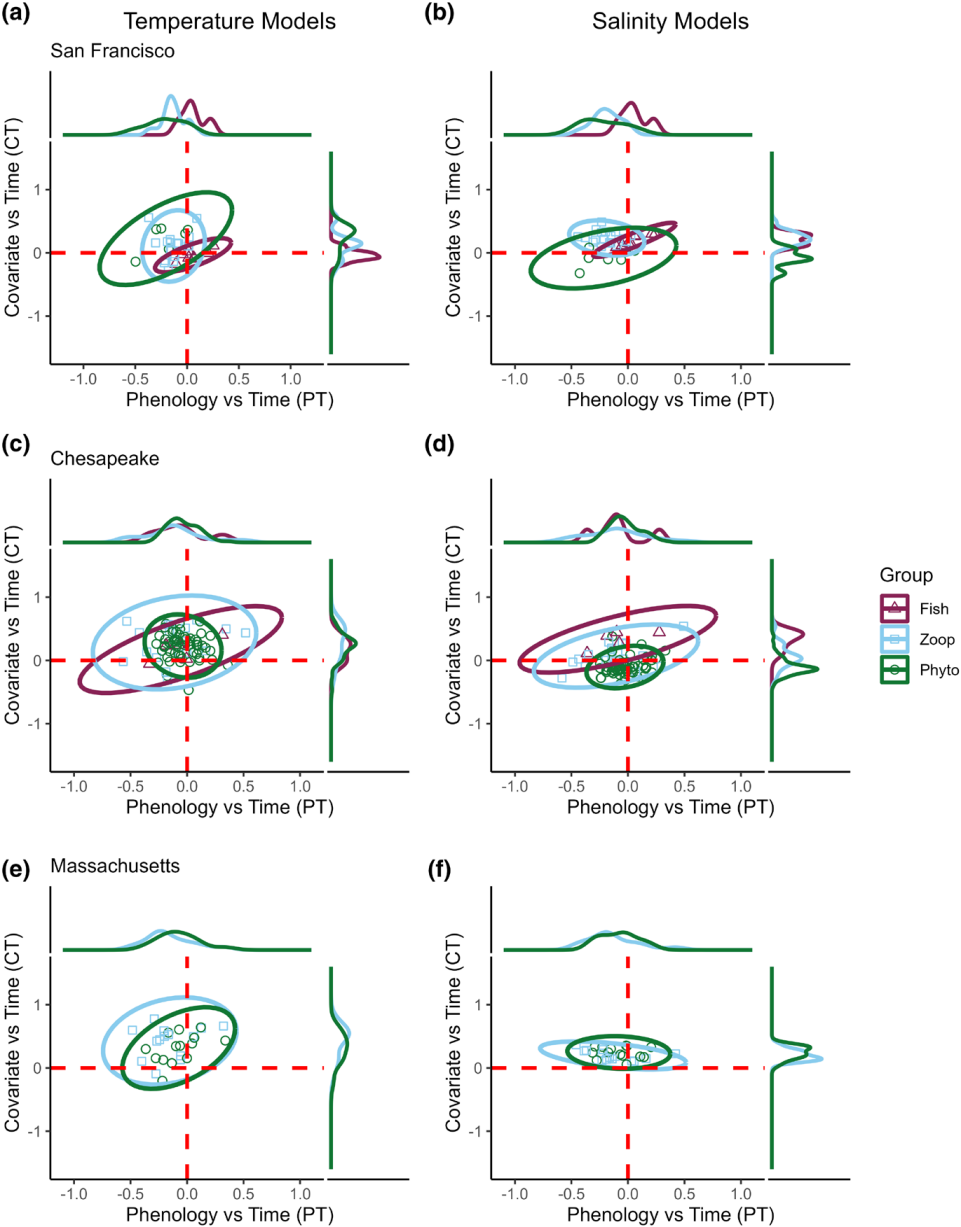
Diversity of phenological shifts and local climate trends, across trophic levels and estuaries. Estimated phenology vs. time slopes (*PT*, *x* axes, values above zero indicate delaying phenologies, values below zero indicate advancing phenologies) and local climate trend slopes (*CT*, *y* axes, values above zero indicates increasing temperature or salinity, below zero indicates decreasing temperature or salinity) are shown for each estuary. Left column: slopes from models estimating annual temperature effects; right column: slopes from models estimating annual salinity effects. For example, a taxon in the top left quadrant of panel A would be advancing its phenology in a warming climate (or in the bottom left quadrant, it would be advancing its phenology in a cooling climate). Similarly, a taxon in the top left quadrant of panel B would be advancing its phenology in an estuary that is increasing in salinity (or in the bottom left quadrant, it would be advancing its phenology in an estuary that is decreasing in salinity). For each estuary and model type, Bayesian 95% confidence ellipses were fitted to visualize the diversity of phenological and local climatic trends experienced by each taxonomic group (Fish, Phytoplankton, Zooplankton). See Figure S6 for more information on how environmental and biological trends are captured in the *PT* and *CT* dimensions.

Bayesian ellipses fitted around the *PT* and *CT* slopes show the diversity of environmental and phenological trends present in each taxonomic level and estuary, and largely confirmed that phenological trends for phytoplankton and zooplankton tended to skew towards advancing peak abundance (Figure 3). However, nuanced patterns emerged. In San Francisco Bay, fishes occupied a narrower trend range, with most of the ellipse indicating delaying *PT* trends. Notably, zooplankton and fishes shared only 21.1% of ellipse area for temperature models and 23.4% of ellipse area for salinity models—indicating diverging phenological and climatic trends. In Chesapeake Bay, all trophic groups were skewed towards advancing phenologies. However, zooplankton exhibited a wider diversity of trends (ellipse area = 1.47 for temp; 0.89 for salinity) compared to fishes (ellipse area = 0.74 for temperature; 0.58 for salinity) and phytoplankton (ellipse area = 0.51 for temperature; 0.33 for salinity). In Massachusetts Bay, zooplankton showed a slightly broader range of phenological and climatic trends (ellipse area = 1.06 for temperature; 0.33 for salinity) than phytoplankton (0.76 for temperature; 0.31 for salinity); however, they shared substantial overlap in trends (96% of their ellipse space).

Overall, we found that phenological shifts were steeper (lower *PT* values, indicating stronger advancements) for zooplankton than for fishes and phytoplankton (*F*_2,10.69_, *p* < 0.001), but this pattern varied by estuary (*F*_3,4.204_, *p* = 0.0062 for the trophic group × estuary interaction term). *PC* slopes, which represent the magnitude and direction of phenological sensitivity to environmental trends, showed that phenology was more sensitive to changes in temperature than to changes in salinity (mean ± SD: 0.154 ± 0.220 for temperature, −0.001 ± 0.180 for salinity; *F*_1,45.573_, *p* < 0.001; Figure S8). Sensitivity also varied across taxonomic groups in ways that were specific to each estuary (*F*_3,4.227_, *p* = 0.006 for the group x estuary interaction term). Collectively, our results suggest that long‐term changes in temperature and salinity often drove phenological shifts, but many different responses were possible—emerging from a combination of diverse local signatures of environmental change, and diverse, taxa‐specific, sensitivities to that change.

### Do changing phenologies track the changing environment?

**FIGURE 4:**
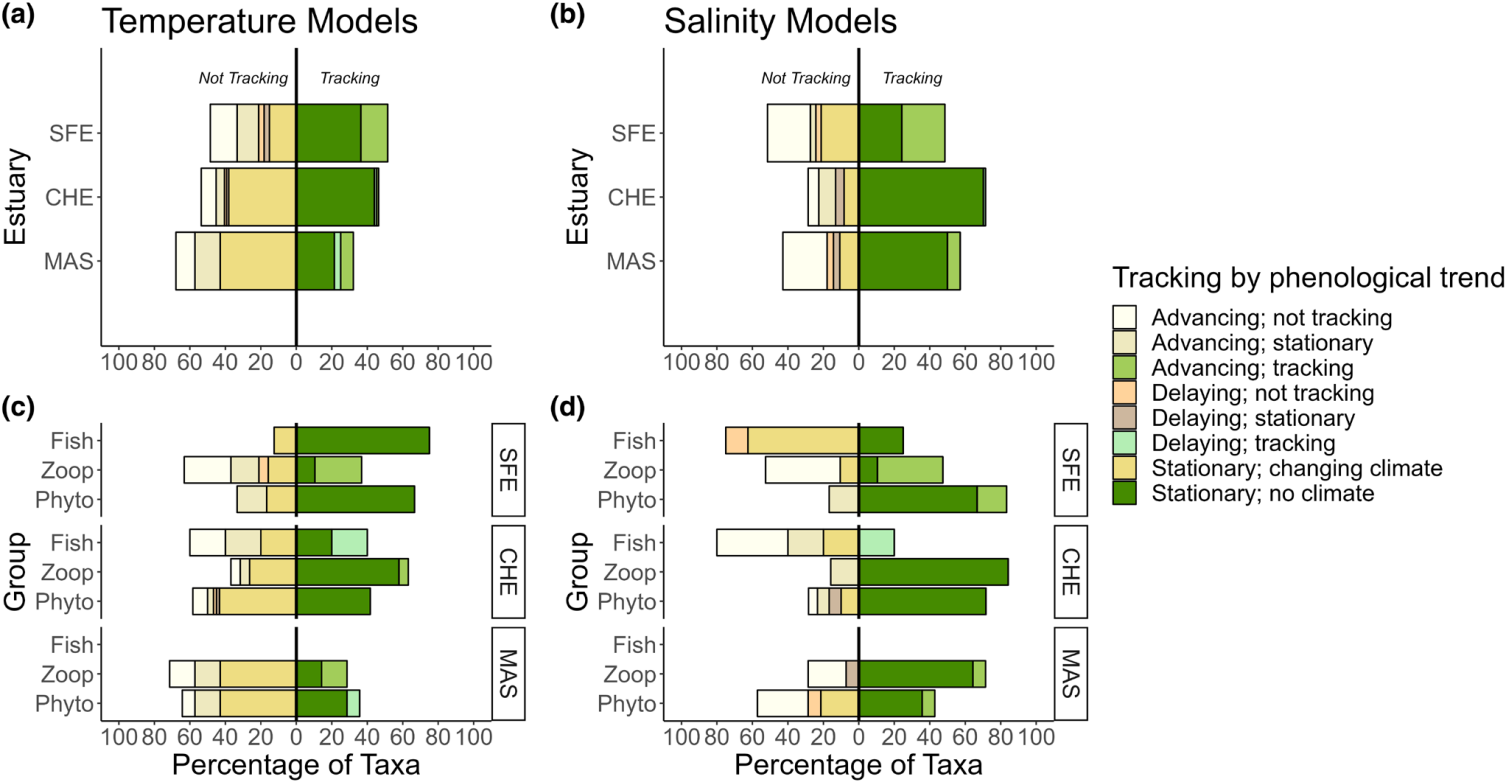
Do changing phenologies track the changing environment? Left column: Proportion of all modelled taxa at each estuary tracking vs. not‐tracking annual temperature (a, c) and annual salinity trends (b, d). For all panels, bars growing towards the left of zero represent taxa not tracking climate trends; bars growing towards the right of 0 represent taxa tracking climate trends. Additionally, shifts are noted as advancing or delaying (see colour legend). We considered taxa tracking their climate those that (1) shifted their phenology, (2) existed within a non‐stationary local climate, and (3) had a significant *PC* slope that was consistent with climatic and phenological trends (e.g., a positive *PC* slope when the taxa is advancing its phenology with a negative *CT* slope). Additionally, organisms that were not shifting their phenologies were considered to be tracking stationary climates. Organisms not tracking climate are broken down into non‐shifting taxa in changing climates (‘Stationary, changing climate’), or taxa without a significant *PC* slope and/or a *PC* slope not consistent with climatic and phenological trends (‘not tracking’).

Taxa at each estuary demonstrated variable capacities to track environmental covariates (32.1–71.4% of taxa tracking). When broken down by taxonomic group, we found that taxa tracked at differential rates for temperature (SFE X^2^_2,13.529_, *p* = 0.001, CHE X^2^_2,6.911_, *p* = 0.03) and salinity in the San Francisco and Chesapeake bays (SFE X^2^_2,33.375_, *p* < 0.001, CHE X^2^_2,39.515_, *p* < 0.001; Figure 4), but not in Masachusetts Bay. The bulk of tracking organisms experienced stable environments, and thus environmentally driven phenological shifts were not expected. In turn, most of the organisms classified as ‘not tracking’ their environments showed stable phenologies despite living in a changing environment. When focusing on the subset of taxa with shifting phenologies, San Francisco Bay taxa tended to track changes in annual salinity more often than changes in temperature. Within those groups, all shifting zooplankton and phytoplankton species were advancing, while fishes tended to delay their phenologies. Conversely, shifting taxa in the Chesapeake and Massachusetts bays tended to track changes in temperature more frequently—with most shifting taxa advancing their timing of peak abundance.

**FIGURE 5:**
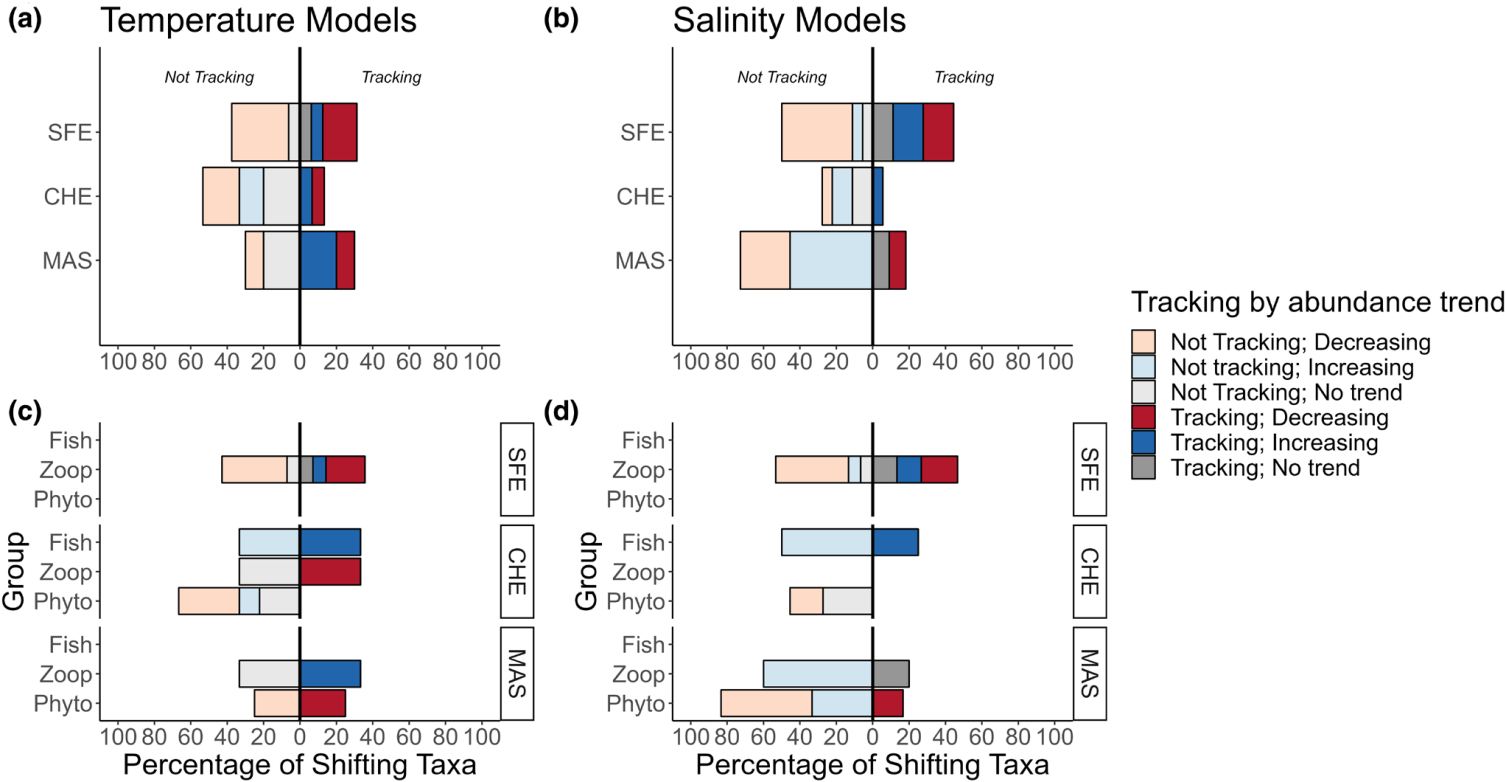
Abundance trends by phenological pattern. Right column: Abundance trends (calculated as a positive or negative correlation with year) of taxa that are shifting phenology with respect to changing annual temperature (a, c) and annual salinity trends (b, d). For all panels, bars growing towards the left of zero represent taxa not tracking climate trends; bars growing towards the right of 0 represent taxa tracking climate trends. We considered taxa tracking their climate those that (1) shifted their phenology, (2) existed within a non‐stationary local climate, and (3) had a significant *PC* slope that was consistent with climatic and phenological trends (e.g., a positive *PC* slope when the taxa is advancing its phenology with a negative *CT* slope).

When we compared the subset of phenologically‐shifting taxa in changing environments to their abundance trends, we found that taxa were often decreasing in abundance despite tracking the environment (X^2^_8,48.329_, *p* < 0.001 for differences in abundance trends that track temperature; X^2^_8,47.34_, *p* < 0.001 for salinity, Figure 5). This pattern was especially pronounced in San Francisco Bay, where 21.43% and 20% of taxa that tracked temperature and salinity, respectively, showed decreasing abundance trends (Figure 5). Substantial variation existed across estuaries and trophic groups, and in some cases the reverse was also true: non‐tracking taxa could exhibit increasing trends. Overall, these results suggest that tracking status and abundance trends were largely decoupled.

### Potential for mismatches in regional food webs

**FIGURE 6:**
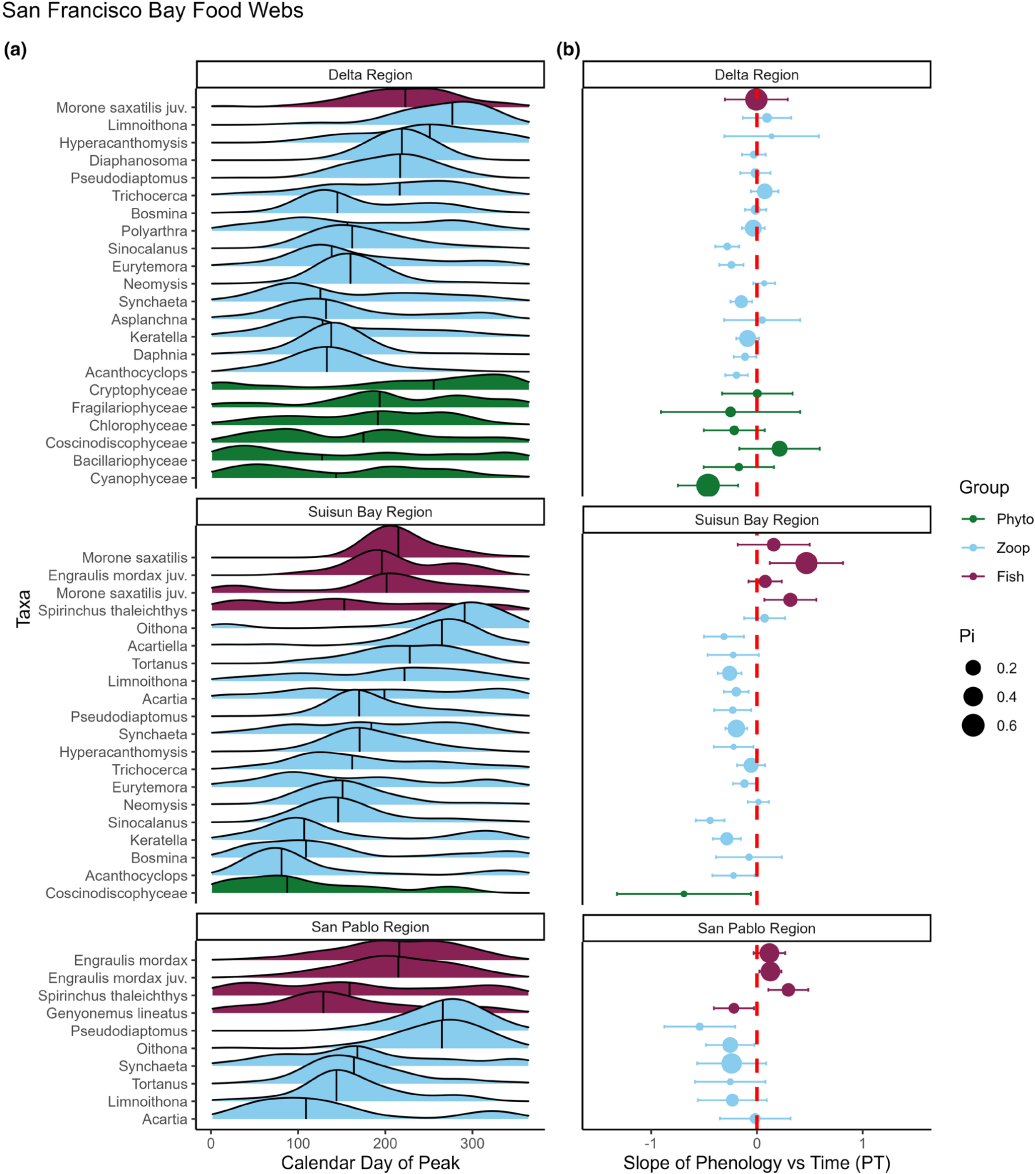
Detailed examination of phenological shifts across relevant taxa of the San Francisco Bay. Local food webs are depicted along the salinity gradient—the Delta is at the low end of the salinity gradient while San Pablo Bay is at the high end (see Figure 2). (a) Distribution of the calendar day of peak abundance for taxa in each regional food web. (b) Individual points represent the maximum likelihood estimate for the slope and associated 95% confidence intervals. Points and CI's to the left of zero are advancing their phenologies, while points and CI's to the right of the zero are delaying their phenologies (colour coded by trophic level, see legend). Point size represents relative mean abundance within the relevant trophic level. The results from our model (panel b) show support for strong phenological response diversity both between (Figure 1a.iii) and within (Figure 1a.iv) trophic levels. We report taxa‐specific phenological trends for each food web in San Francisco Bay as sampling in this region is the most complete and represents the only dataset that incorporates all three taxonomic groups for most of the time series.

We found similarly high variances between taxonomic groups, as each group consistently displayed a relatively broad portfolio of phenological trends (*F*_2,2.548_, *p* = 0.08, Figure S7). These patterns support those illustrated in response type iv of Figure 1a. However, because diverging phenologies may only lead to a trophic mismatch if predator and prey co‐occur locally, we examined the potential for mismatches at a finer spatial scale within each system. We found that historical ranges of peak abundance within trophic levels varied, but many taxa peaked around similar points within the year and thus might have historically overlapped through space and time. In San Francisco Bay, we found 15 species shifting their phenologies. Among these, 15 zooplankton taxa in 3 ecologically‐distinct regions displayed advancing phenologies, while fishes displayed both advancing (*n* = 1) or delaying phenologies (*n* = 2 in 2 regions). Two phytoplankton taxa showed a significant advancing trend. In this system, Suisun Bay is in an intermediate salinity zone bookended by the fresher Delta and the saltier San Pablo Bay. Across regions in San Francisco Bay, Suisun Bay showed the most shifts (*n* = 12) and the divergence between fishes and zooplankton was especially pronounced (Figure 6). Moreover, we found that phenological trends were significantly divergent between trophic groups in Suisun Bay (*F*_2,24.17_, *p* < 0.001) and San Pablo Bay (*F*_1,7.826_, *p* = 0.02), but not in the Delta (*F*_2,0.889_, *p* = 0.428). These trends indicate a high potential for trophic mismatches in these two regions (see Figure 1a, response type iii). Additionally, we found similar levels of trend divergence in the Chesapeake and Massachusetts bays (see full results on these systems in Supporting Information S6). Overall, these results illustrate that despite the wide variation in environmental trends and phenological responses to those trends, ample potential for trophic mismatches exists in local food webs.

## DISCUSSION

Despite growing evidence of phenological shifts in the context of global climate change (Vitasse et al., 2022), research comprising whole food webs remains scarce (but see Donnelly et al., 2011, Visser & Gienapp, 2019). Here, we investigated phenological shifts and their drivers within and across trophic levels in three major US estuaries. We found that many taxa shifted their phenology (timing of peak abundance), and that these shifts were largely towards earlier peaks. Additionally, the magnitude of phenological trends and climatic sensitivities diverged between trophic groups and estuaries. Despite these patterns, many taxa with shifting phenology did not track changing environments, and taxonomic groups often tracked salinity trends at different rates. Finally, while phenological trend diversity was consistently high across trophic levels (i.e., response Figure 1a, type iv), divergent patterns of phenological change and climatic drivers often scaled down to more localized food webs (Figure 1a, response type iii), illustrating high potential for trophic mismatch. Consequently, widespread phenological change may be poised to disrupt estuarine food‐web dynamics across broad spatial scales.

### Sensitivity to environmental drivers varies within and across trophic levels

We found that many taxa shifted their phenology, but sensitivities varied in magnitude and direction within and across trophic levels. At the population scale, the sensitivity of a given taxon to climate‐driven phenological shifts has implications for migratory patterns, distribution shifts, and demography (Hare et al., 2016; Thackeray et al., 2016). In our case, plankton displayed high sensitivity to temperature changes. However, shifting taxa in San Francisco Bay frequently tracked changes in salinity. Prolonged multi‐year droughts in California severely diminished freshwater inputs, resulting in increased salinity over time (Hutton et al., 2016). While temperature has been comparatively stable in this system, planktonic taxa retain high sensitivity to temperature fluctuations, suggesting that future warming or cooling could drive phenological shifts more strongly than current observations. Shifts in zooplankton phenology have been documented throughout San Francisco Bay due to various factors, including temperature, salinity, and bivalve invasions (Cloern & Jassby, 2012; Merz et al., 2016). However, the specific impact of these shifts on community composition and structure remains largely unknown. In Chesapeake and Massachusetts bays, changing temperatures seemed to influence more shifts. Specifically, warming trends in the Gulf of Maine and widespread warming throughout the Atlantic might strongly impact communities in Massachusetts and Chesapeake Bays, respectively (Hinson et al., 2022; Staudinger et al., 2019). These overarching environmental conditions lead to similar levels of sensitivity and phenological response between zooplankton and phytoplankton.

Our findings revealed that fishes in Chesapeake Bay exhibited a greater sensitivity to changes in temperature than those in the San Francisco Bay. Temperature ranges in the Chesapeake Bay are highly variable, and often lead to decreased residency time for fishes within the system (Schonfeld et al., 2022). For instance, temperature has a strong influence on the timing of Striped Bass (*Morone saxatilis*, Walbaum) breeding behaviours in the Chesapeake (Peer & Miller, 2014). Comparatively, temperature patterns experienced by fishes in San Francisco Bay are more stable, but changes in salinity (seasonal and interannual) are pronounced. Although salinity fluctuations, largely driven by Delta outflow, influence fish migration timing in the San Francisco Bay (e.g., Goertler et al., 2021), we observed that many fishes failed to track long‐term salinity trends. This pattern could indicate weaker (or inconsistent) responses of these species to long‐term hydrologic trends; or the fact that behavioural patterns not assessed by our models (i.e., migration timings) might mask abundance trends. On another note, because migration phenology is often linked to body size in fish populations (Jonsson et al., 2017), direct exploitation (often size‐dependent) might influence phenological dynamics by altering population size spectra. Overall, a combination of local climatic variation and anthropogenic impacts might influence phenological plasticity across a species distribution range (Ovaskainen et al., 2013).

The differential phenological sensitivities and environmental tracking observed between fishes and plankton can be attributed to their distinct life histories and physiologies. Fishes are longer‐lived and more mobile (Beitinger et al., 2000), and their extended lifespan may allow them to acclimate to a wider range of temperature conditions. However, metabolic scope changes with ontogeny, and populations can be affected if environmental conditions impact key life stages (Wingate & Secor, 2008). Additionally, higher mobility can enable fishes to actively seek refuge in response to deleterious environmental conditions (Boucek et al., 2017). Indeed, decreased habitat use within the estuary might obfuscate abundance trends if previously resident species are sampled less frequently (Schonfeld et al., 2022). In contrast, shorter life cycles might make plankton more susceptible to fine‐scale temperature changes that can directly impact their abundances. Furthermore, while fishes may exhibit phenological shifts as a response to environmental changes, plankton populations often have more limited tolerances that could instead result in population declines (Qasim et al., 1972). These disparities in sensitivities and responses to environmental drivers may disrupt interactions between taxa if phenological changes become sufficiently pronounced.

### Rates and directions of phenological change might alter food web dynamics

Our models identified taxa across all trophic levels that were shifting their phenologies—with zooplankton exhibiting the most pronounced and highest proportion of shifts (Figures 3, 4). If phenological shifts of planktonic taxa outpace those of their predators, it could disrupt food‐web stability by limiting the availability of food resources for consumers (match–mismatch hypothesis) or by increasing predation pressure through trophic release (Jachowski et al., 2020). Under extreme levels, unchecked planktonic population growth could exacerbate harmful algal blooms. Furthermore, a sufficiently pronounced shift of a top predator could introduce them into a new pool of naive prey and cause cascading ecological effects to the food web. However, the ultimate effects depend on baseline synchrony (Downing et al., 2008)—and our models do not explicitly account for historical species overlap and interaction strength. In San Francisco Bay, zooplankton groups historically peaked at various times throughout the year (Figure 6a). However, our models indicate that many zooplankters in this system are advancing their phenologies despite varied ‘starting points’. While rates of phenological change are not uniform, the overall pattern of advancement might be indicative of an assemblage striving to maintain a historical portfolio of temporal partitioning. Additionally, dispersion or temporal variability in peak dates further reflects the ability of a given species to track fluctuations in the environment, and thus may be linked to population persistence. Similarly, the high dispersion of trends we observed at the community level underlines that species‐rich assemblages may be more stable than species‐poor ones, and that predators may benefit from relying on prey taxa that peak across wide temporal windows. While a diversity of trends might allow other species to occupy niches left vacant by shifting taxa (Reeb et al., 2020), a phenological shift by keystone species might produce substantial consequences (Rather et al., 2018). For example, Right Whales (*Eubalaena* sp.) often feed on *Calanus* during their migration along the eastern coast of North America (Baumgartner et al., 2003). Our models showed that *Calanus* appears to be shifting its phenology within Massachusetts Bay, and previous work indicates these shifts might already be propagating up the food chain (Charif et al., 2020; Pendleton et al., 2022).

Beyond advancing phenology, we also found several delays—particularly among fishes. The Longfin Smelt (*Spirinchus thaleichthys*, Ayres) in the San Francisco Bay and the Atlantic Croaker (*Micropogonias undulatus*, Linneaus) in the Chesapeake Bay both displayed system‐wide delays. Additionally, juvenile Northern Anchovy (*Engraulis mordax*, Girard) are delaying in the Suisun and San Pablo Bays subregions of San Francisco Bay. Of note, the Striped Bass is trending towards delay in the San Francisco Bay but trending towards advance in the Chesapeake. These divergent phenological patterns highlight the role of local‐scale climatic signatures on population dynamics. Notably, diverse responses did not prevent widespread population declines observed across our study systems, which likely reflect high local extinction risk (Figure 5) and an increased risk of trophic mismatches (Chevillot et al., 2017). Further research is needed to understand the impacts of diverging predator–prey phenologies in the context of potential versus realized trophic links, and how predator omnivory may decrease such risk.

### Concluding remarks

Detection and attribution of phenological shifts is often challenged by the scarcity of multi‐trophic time series data. Here, we leveraged long‐term, community‐scale monitoring data that facilitated comparing phenology across trophic levels while maintaining local climate context (Cohen et al., 2018). However, our approach is not without limitations. First, our analyses were limited by the monthly frequency of the monitoring data, which can be relatively coarse for species with fast boom‐and‐bust dynamics and short‐lived peaks; as well as for species without strongly defined unimodal peaks (e.g., Striped Bass in the Chesapeake Bay). More frequent sampling and alternate modelling strategies would allow understanding finer changes not only in timing of the absolute peak but also in the length of high‐abundance windows, which could contract or expand as a result of climate forcing (Rogers & Dougherty, 2019). Second, our models do not account for temporal autocorrelation. Though we found that the impact of any autocorrelation in the phenological and environmental time series was low (Supporting Information S4), time‐series methods may provide more nuanced analyses. Third, we focused our analyses on taxa with high abundances and detection rates to enhance confidence in model estimates. Thus, we did not model many taxa that are often present in the study estuaries that were not targeted by sampling gear and protocols (e.g., ichthyoplankton). Fourth, the trivariate structure of our models examined one climatic variable at a time (temperature or salinity), but multiple interacting stressors often drive ecological structure and function (Breitburg et al., 1998). Finally, biological invasions are a key concern in estuarine systems (Dijkstra et al., 2011) and have the potential to influence phenological shifts (Colautti et al., 2017). Research that explicitly incorporates biotic interactions alongside abiotic drivers would provide a more comprehensive understanding of the mechanisms driving phenological change.

The transitional nature of estuaries makes them uniquely vulnerable to global change stressors (Lauchlan & Nagelkerken, 2020). We found that many organisms are shifting phenologies, but because many changing phenologies do not track the changing environment, there is a strong potential for disruption of estuarine food‐web dynamics. However, the diversity of responses we observed within trophic levels introduces significant uncertainty regarding the overall impact that divergent phenological trends will have on community dynamics. A better understanding of the mechanisms driving phenological shifts, and the limits of organisms to shift their phenology in response to changing climates, will help inform more effective conservation and management strategies for these vulnerable ecosystems.

## AUTHOR CONTRIBUTIONS

RJF conceptualized the study, compiled data, fitted statistical models, analysed and visualized modelling outputs, interpreted results, and drafted the manuscript. SMC and AR supervised the grant, conceptualized the study, interpreted results, and edited the manuscript. DDC conceptualized the study and edited the manuscript. RJL provided data and edited the manuscript.

## Supporting information




Data S1.

## Data Availability

Data, metadata, and R code necessary to reproduce model results, analyses, and figures are accessible via Data Dryad https://doi.org/10.6078/D1QT4K and Zenodo: https://doi.org/10.5281/zenodo.10093054. Raw abundance time series data can be accessed via their collecting agencies: California Department of Fish and Wildlife, Massachusetts Water Resources Authority, Chesapeake Bay Program, and the Virginia Institute of Marine Science. See ‘Data provenance’ section of associated metadata for a full list of data sources and contact information.

## References

[ele14441-bib-0001] Apple, J.K. , Del Giorgio, P.A. & Kemp, W.M. (2006) Temperature regulation of bacterial production, respiration, and growth efficiency in a temperate salt‐marsh estuary. Aquatic Microbial Ecology, 43(3), 243–254.

[ele14441-bib-0002] Asch, R.G. , Stock, C.A. & Sarmiento, J.L. (2019) Climate change impacts on mismatches between phytoplankton blooms and fish spawning phenology. Global Change Biology, 25(8), 2544–2559. 31152499 10.1111/gcb.14650

[ele14441-bib-0003] Baumgartner, M.F. , Cole, T.V. , Campbell, R.G. , Teegarden, G.J. & Durbin, E.G. (2003) Associations between North Atlantic right whales and their prey, Calanus finmarchicus, over diel and tidal time scales. Marine Ecology Progress Series, 264, 155–166.

[ele14441-bib-0004] Beck, M.W. , Heck, K.L. , Able, K.W. , Childers, D.L. , Eggleston, D.B. , Gillanders, B.M. et al. (2001) The identification, conservation, and management of estuarine and marine nurseries for fish and invertebrates: a better understanding of the habitats that serve as nurseries for marine species and the factors that create site‐specific variability in nursery quality will improve conservation and management of these areas. Bioscience, 51(8), 633–641.

[ele14441-bib-0005] Beitinger, T.L. , Bennett, W.A. & McCauley, R.W. (2000) Temperature tolerances of north American freshwater fishes exposed to dynamic changes in temperature. Environmental Biology of Fishes, 58, 237–275.

[ele14441-bib-0006] Boucek, R.E. , Heithaus, M.R. , Santos, R. , Stevens, P. & Rehage, J.S. (2017) Can animal habitat use patterns influence their vulnerability to extreme climate events? An estuarine sportfish case study. Global Change Biology, 23(10), 4045–4057. 28593715 10.1111/gcb.13761

[ele14441-bib-0007] Breitburg, D.L. , Baxter, J.W. , Hatfield, C.A. , Howarth, R.W. , Jones, C.G. , Lovett, G.M. et al. (1998) Understanding effects of multiple stressors: ideas and challenges. In: Successes, limitations, and frontiers in ecosystem science, New York, NY: Springer, pp. 416–431.

[ele14441-bib-0008] Brown, C.J. (2016) Ecological and methodological drivers of species' distribution and phenology responses to climate change. Global Change Biology, 22, 1548–1560. 26661135 10.1111/gcb.13184

[ele14441-bib-0009] CBP . (2023) Chesapeake Bay Program . https://www.chesapeakebay.net/

[ele14441-bib-0010] CDFW . (2023a) California Department of Fish and Wildlife San Francisco Bay study: Long‐term fish and water quality monitoring data . https://filelib.wildlife.ca.gov/Public/BayStudy/

[ele14441-bib-0011] CDFW . (2023b) Interagency Ecological Monitoring Program . https://iep.ca.gov/Science‐Synthesis‐Service/Monitoring‐Programs/EMP

[ele14441-bib-0012] Charif, R.A. , Shiu, Y. , Muirhead, C.A. , Clark, C.W. , Parks, S.E. & Rice, A.N. (2020) Phenological changes in North Atlantic right whale habitat use in Massachusetts Bay. Global Change Biology, 26(2), 734–745. 31729818 10.1111/gcb.14867

[ele14441-bib-0013] Chevillot, X. , Drouineau, H. , Lambert, P. , Carassou, L. , Sautour, B. & Lobry, J. (2017) Toward a phenological mismatch in estuarine pelagic food web? PLoS One, 12(3), 173752. 10.1371/journal.pone.0173752 PMC5371289 28355281

[ele14441-bib-0014] Chmura, H.E. , Kharouba, H.M. , Ashander, J. , Ehlman, S.M. , Rivest, E.B. & Yang, L.H. (2019) The mechanisms of phenology: the patterns and processes of phenological shifts. Ecological Monographs, 89(1), 1337.

[ele14441-bib-0015] Cloern, J.E. & Jassby, A.D. (2012) Drivers of change in estuarine‐coastal ecosystems: discoveries from four decades of study in San Francisco Bay. Reviews of Geophysics, 50(4), 1–33.

[ele14441-bib-0016] Cohen, J.M. , Lajeunesse, M.J. & Rohr, J.R. (2018) A global synthesis of animal phenological responses to climate change. Nature Climate Change, 8(3), 224–228.

[ele14441-bib-0017] Colautti, R.I. , Ågren, J. & Anderson, J.T. (2017) Phenological shifts of native and invasive species under climate change: insights from the Boechera–Lythrum model. Philosophical Transactions of the Royal Society, B: Biological Sciences, 372(1712), 20160032. 10.1098/rstb.2016.0032 PMC5182428 27920377

[ele14441-bib-0018] Colombano, D.D. , Manfree, A.D. , Teejay, A.O. , Durand, J.R. & Moyle, P.B. (2020) Estuarine‐terrestrial habitat gradients enhance nursery function for resident and transient fishes in the San Francisco estuary. Marine Ecology Progress Series, 637, 141–157.

[ele14441-bib-0019] Dijkstra, J.A. , Westerman, E.L. & Harris, L.G. (2011) The effects of climate change on species composition, succession and phenology: a case study. Global Change Biology, 17(7), 2360–2369.

[ele14441-bib-0020] Donnelly, A. , Caffarra, A. & O'Neill, B.F. (2011) A review of climate‐driven mismatches between interdependent phenophases in terrestrial and aquatic ecosystems. International Journal of Biometeorology, 55, 805–817. 21509461 10.1007/s00484-011-0426-5

[ele14441-bib-0021] Downing, A.L. , Brown, B.L. , Perrin, E.M. , Keitt, T.H. & Leibold, M.A. (2008) Environmental fluctuations induce scale‐dependent compensation and increase stability in plankton ecosystems. Ecology, 89(11), 3204–3214. 31766790 10.1890/07-1652.1

[ele14441-bib-0022] Duchenne, F. , Martin, G. & Porcher, E. (2021) European plants lagging behind climate change pay a climatic debt in the north, but are favoured in the south. Ecology Letters, 24(6), 1178–1186. 33750013 10.1111/ele.13730

[ele14441-bib-0023] Farnsworth, E.J. , Nunez‐Farfan, J. , Careaga, S.A. & Bazzaz, F.A. (1995) Phenology and growth of three temperate forest life forms in response to artificial soil warming. Journal of Ecology, 83, 967–977.

[ele14441-bib-0024] Fournier, R.J. , Colombano, D.D. , Latour, R.J. , Carlson, S.M. & Ruhi, A. (2023) Long‐term data reveal widespread phenological change across major U.S. estuaries . 10.6078/D1QT4K PMC11686945 39738977

[ele14441-bib-0025] Ghalambor, C.K. , Gross, E.S. , Grosholtz, E.D. , Jeffries, K.M. , Largier, J.K. , McCormick, S.D. et al. (2021) Ecological effects of climate‐driven salinity variation in the San Francisco estuary: can we anticipate and manage the coming changes? San Francisco Estuary and Watershed Science, 19(2), 1–30.

[ele14441-bib-0026] Goertler, P. , Mahardja, B. & Sommer, T. (2021) Striped bass (Morone saxatilis) migration timing driven by estuary outflow and sea surface temperature in the San Francisco Bay‐Delta, California. Scientific Reports, 11(1), 1510. 33452283 10.1038/s41598-020-80517-5 PMC7810903

[ele14441-bib-0027] Gordo, O. (2007) Why are bird migration dates shifting? A review of weather and climate effects on avian migratory phenology. Climate Research, 35, 37–58.

[ele14441-bib-0028] Guinder, V.A. , Molinero, J.C. , López Abbate, C.M. , Berasategui, A.A. , Popovich, C.A. , Spetter, C.V. et al. (2017) Phenological changes of blooming diatoms promoted by compound bottom‐up and top‐down controls. Estuaries and Coasts, 40, 95–104.

[ele14441-bib-0029] Hare, J.A. , Morrison, W.E. , Nelson, M.W. , Stachura, M.M. , Teeters, E.J. , Griffis, R.B. et al. (2016) A vulnerability assessment of fish and invertebrates to climate change on the northeast US continental shelf. PLoS One, 11(2), 146756. 10.1371/journal.pone.0146756 PMC4739546 26839967

[ele14441-bib-0030] Harrison, T.D. & Whitfield, A.K. (2006) Temperature and salinity as primary determinants influencing the biogeography of fishes in south African estuaries. Estuarine, Coastal and Shelf Science, 66(1–2), 335–345.

[ele14441-bib-0031] Hinson, K.E. , Friedrichs, M.A. , St‐Laurent, P. , Da, F. & Najjar, R.G. (2022) Extent and causes of Chesapeake Bay warming. JAWRA Journal of the American Water Resources Association, 58(6), 805–825.

[ele14441-bib-0032] Hutton, P.H. , Rath, J.S. , Chen, L. , Ungs, M.J. & Roy, S.B. (2016) Nine decades of salinity observations in the San Francisco Bay and Delta: modeling and trend evaluations. Journal of Water Resources Planning and Management, 142(3), 4015069.

[ele14441-bib-0033] Incze, L.S. , Lutz, R.A. & Watling, L. (1980) Relationships between effects of environmental temperature and seston on growth and mortality of Mytilus edulis in a temperate northern estuary. Marine Biology, 57, 147–156.

[ele14441-bib-0034] Jachowski, D.S. , Butler, A. , Eng, R.Y. , Gigliotti, L. , Harris, S. & Williams, A. (2020) Identifying mesopredator release in multi‐predator systems: a review of evidence from North America. Mammal Review, 50(4), 367–381.

[ele14441-bib-0035] Jackson, A. , Parnell, A. & Jackson, M.A. (2019) Package ‘SIBER.’ . R Package Version, 2(4).

[ele14441-bib-0036] Jonsson, B. , Jonsson, M. & Jonsson, N. (2017) Influences of migration phenology on survival are size‐dependent in juvenile Atlantic salmon (Salmo salar). Canadian Journal of Zoology, 95(8), 581–587.

[ele14441-bib-0037] Koeller, P. , Fuentes‐Yaco, C. , Platt, T. , Sathyendranath, S. , Richards, A. , Ouellet, P. et al. (2009) Basin‐scale coherence in phenology of shrimps and phytoplankton in the North Atlantic Ocean. Science, 324(5928), 791–793. 19423827 10.1126/science.1170987

[ele14441-bib-0038] Kristiansen, I. , Gaard, E. , Hátún, H. , Jónasdóttir, S. & Ferreira, A.S.A. (2016) Persistent shift of Calanus spp. in the southwestern Norwegian Sea since 2003, linked to ocean climate. ICES Journal of Marine Science, 73(5), 1319–1329.

[ele14441-bib-0039] Kromkamp, J.C. & Engeland, T. (2010) Changes in phytoplankton biomass in the Western Scheldt estuary during the period 1978–2006. Estuaries and Coasts, 33, 270–285.

[ele14441-bib-0040] Lane, R.R. , Day, J.W., Jr. , Marx, B.D. , Reyes, E. , Hyfield, E. & Day, J.N. (2007) The effects of riverine discharge on temperature, salinity, suspended sediment and chlorophyll a in a Mississippi delta estuary measured using a flow‐through system. Estuarine, Coastal and Shelf Science, 74(1–2), 145–154.

[ele14441-bib-0041] Langan, J.A. , Puggioni, G. , Oviatt, C.A. , Henderson, M.E. & Collie, J.S. (2021) Climate alters the migration phenology of coastal marine species. Marine Ecology Progress Series, 660, 1–18.

[ele14441-bib-0042] Latour, R.J. , Gartland, J. & Bonzek, C.F. (2023) Design and redesign of a bottom trawl survey in Chesapeake Bay, USA. Frontiers in Marine Science, 10, 1–15.

[ele14441-bib-0043] Lauchlan, S.S. & Nagelkerken, I. (2020) Species range shifts along multistressor mosaics in estuarine environments under future climate. Fish and Fisheries, 21(1), 32–46.

[ele14441-bib-0044] Leathers, K. , Herbst, D. , de Mendoza, G. , Doerschlag, G. & Ruhi, A. (2024) Climate change is poised to alter mountain stream ecosystem processes via organismal phenological shifts. Proceedings of the National Academy of Sciences, 121(14), e2310513121. 10.1073/pnas.2310513121 PMC10998557 38498724

[ele14441-bib-0045] Mann, M.E. & Lees, J.M. (1996) Robust estimation of background noise and signal detection in climatic time series. Climatic Change, 33(3), 409–445.

[ele14441-bib-0046] Marques, S.C. , Azeiteiro, U.M. , Marques, J.C. , Neto, J.M. & Pardal, M.Â. (2006) Zooplankton and ichthyoplankton communities in a temperate estuary: spatial and temporal patterns. Journal of Plankton Research, 28(3), 297–312.

[ele14441-bib-0047] McQueen, K. & Marshall, C.T. (2017) Shifts in spawning phenology of cod linked to rising sea temperatures. ICES Journal of Marine Science, 74(6), 1561–1573.

[ele14441-bib-0048] Merz, J.E. , Bergman, P.S. , Simonis, J.L. , Delaney, D. , Pierson, J. & Anders, P. (2016) Long‐term seasonal trends in the prey community of Delta smelt (*Hypomesus transpacificus*) within the Sacramento‐san Joaquin Delta, California. Estuaries and Coasts, 39, 1526–1536.

[ele14441-bib-0049] Mori, A.S. , Furukawa, T. & Sasaki, T. (2013) Response diversity determines the resilience of ecosystems to environmental change. Biological Reviews, 88(2), 349–364. 23217173 10.1111/brv.12004

[ele14441-bib-0050] MWRA . (2023) Massachusetts Water Resources Authority Water Column Monitoring Program . https://www.mwra.com/

[ele14441-bib-0051] Otero, J. , L'Abée‐Lund, J.H. , Castro‐Santos, T. , Leonardsson, K. , Storvik, G.O. , Jonsson, B. et al. (2014) Basin‐scale phenology and effects of climate variability on global timing of initial seaward migration of Atlantic salmon (Salmo salar). Global Change Biology, 20(1), 61–75. 23966281 10.1111/gcb.12363

[ele14441-bib-0052] Ovaskainen, O. , Skorokhodova, S. , Yakovleva, M. , Sukhov, A. , Kutenkov, A. , Kutenkova, N. et al. (2013) Community‐level phenological response to climate change. Proceedings of the National Academy of Sciences, 110(33), 13434–13439. 10.1073/pnas.1305533110 PMC3746850 23901098

[ele14441-bib-0053] Peer, A.C. & Miller, T.J. (2014) Climate change, migration phenology, and fisheries management interact with unanticipated consequences. North American Journal of Fisheries Management, 34(1), 94–110.

[ele14441-bib-0054] Pendleton, D.E. , Tingley, M.W. , Ganley, L.C. , Friedland, K.D. , Mayo, C. , Brown, M.W. et al. (2022) Decadal‐scale phenology and seasonal climate drivers of migratory baleen whales in a rapidly warming marine ecosystem. Global Change Biology, 28(16), 4989–5005. 35672922 10.1111/gcb.16225 PMC9541444

[ele14441-bib-0055] Post, E. & Forchhammer, M.C. (2008) Climate change reduces reproductive success of an Arctic herbivore through trophic mismatch. Philosophical Transactions of the Royal Society, B: Biological Sciences, 363(1501), 2367–2373. 10.1098/rstb.2007.2207 PMC2606787 18006410

[ele14441-bib-0056] Qasim, S.Z. , Bhattathiri, P.M.A. & Devassy, V.P. (1972) The influence of salinity on the rate of photosynthesis and abundance of some tropical phytoplankton. Marine Biology, 12, 200–206.

[ele14441-bib-0057] Quinn, G.P. & Keough, M.J. (2002) Experimental design and data analysis for biologists. Cambridge: Cambridge University Press.

[ele14441-bib-0058] Rafferty, N.E. , CaraDonna, P.J. & Bronstein, J.L. (2015) Phenological shifts and the fate of mutualisms. Oikos, 124(1), 14–21. 25883391 10.1111/oik.01523 PMC4396844

[ele14441-bib-0059] Rasmussen, N.L. & Rudolf, V.H. (2016) Individual and combined effects of two types of phenological shifts on predator–prey interactions. Ecology, 97(12), 3414–3421. 27912001 10.1002/ecy.1578

[ele14441-bib-0060] Rather, R.N. , Wani, A.A. , Kashtwari, M. & Beigh, Z.A. (2018) Phenological shifts due to climate change and the associated conservation threats. Climate Change, 4(13), 80–86.

[ele14441-bib-0061] Reeb, R.A. , Acevedo, I. , Heberling, J.M. , Isaac, B. & Kuebbing, S.E. (2020) Nonnative old‐field species inhabit early season phenological niches and exhibit unique sensitivity to climate. Ecosphere, 11(8), 3217.

[ele14441-bib-0062] Rogers, L.A. & Dougherty, A.B. (2019) Effects of climate and demography on reproductive phenology of a harvested marine fish population. Global Change Biology, 25(2), 708–720. 30430699 10.1111/gcb.14483

[ele14441-bib-0063] Satterthwaite, W.H. , Carlson, S.M. , Allen‐Moran, S.D. , Vincenzi, S. , Bograd, S.J. & Wells, B.K. (2014) Match‐mismatch dynamics and the relationship between ocean‐entry timing and relative ocean recoveries of Central Valley fall run Chinook salmon. Marine Ecology Progress Series, 511, 237–248.

[ele14441-bib-0064] Scanes, E. , Scanes, P.R. & Ross, P.M. (2020) Climate change rapidly warms and acidifies Australian estuaries. Nature Communications, 11(1), 1803. 10.1038/s41467-020-15550-z PMC7156424 32286277

[ele14441-bib-0065] Schonfeld, A.J. , Gartland, J. & Latour, R.J. (2022) Spatial differences in estuarine utilization by seasonally resident species in mid‐Atlantic bight, USA. Fisheries Oceanography, 31(6), 615–628.

[ele14441-bib-0066] Simenstad, C.A. , Fresh, K.L. & Salo, E.O. (1982) The role of Puget Sound and Washington coastal estuaries in the life history of Pacific salmon: an unappreciated function. In: Estuarine comparisons. Cambridge, MA: Elsevier, pp. 343–364.

[ele14441-bib-0067] Staudinger, M.D. , Mills, K.E. , Stamieszkin, K. , Record, N.R. , Hudak, C.A. , Allyn, A. et al. (2019) It's about time: a synthesis of changing phenology in the Gulf of Maine ecosystem. Fisheries Oceanography, 28(5), 532–566. 31598058 10.1111/fog.12429 PMC6774335

[ele14441-bib-0068] Tang, J. , Körner, C. , Muraoka, H. , Piao, S. , Shen, M. , Thackeray, S.J. et al. (2016) Emerging opportunities and challenges in phenology: a review. Ecosphere, 7(8), 1436.

[ele14441-bib-0069] Thackeray, S.J. , Henrys, P.A. , Hemming, D. , Bell, J.R. , Botham, M.S. , Burthe, S. et al. (2016) Phenological sensitivity to climate across taxa and trophic levels. Nature, 535(7611), 241–245. 27362222 10.1038/nature18608

[ele14441-bib-0070] Thakur, M.P. (2020) Climate warming and trophic mismatches in terrestrial ecosystems: the green–brown imbalance hypothesis. Biology Letters, 16(2), 20190770.

[ele14441-bib-0071] Thomas, A.C. , Pershing, A.J. , Friedland, K.D. , Nye, J.A. , Mills, K.E. , Alexander, M.A. et al. (2017) Seasonal trends and phenology shifts in sea surface temperature on the north American northeastern continental shelf. Elementa: Science of the Anthropocene, 5, 48.

[ele14441-bib-0072] Varpe, Ø. & Fiksen, Ø. (2010) Seasonal plankton–fish interactions: light regime, prey phenology, and herring foraging. Ecology, 91(2), 311–318. 20391994 10.1890/08-1817.1

[ele14441-bib-0073] Viechtbauer, W. (2010) Conducting meta‐analyses in R with the metafor package. Journal of Statistical Software, 36, 1–48.

[ele14441-bib-0074] Visser, M.E. & Gienapp, P. (2019) Evolutionary and demographic consequences of phenological mismatches. Nature Ecology & Evolution, 3(6), 879–885. 31011176 10.1038/s41559-019-0880-8 PMC6544530

[ele14441-bib-0075] Vitasse, Y. , Baumgarten, F. , Zohner, C.M. , Rutishauser, T. , Pietragalla, B. , Gehrig, R. et al. (2022) The great acceleration of plant phenological shifts. Nature Climate Change, 12(4), 300–302.

[ele14441-bib-0076] Wingate, R.L. & Secor, D.H. (2008) Effects of winter temperature and flow on a summer‐fall nursery fish assemblage in the Chesapeake Bay, Maryland. Transactions of the American Fisheries Society, 137(4), 1147–1156.

[ele14441-bib-0078] Woodward, G. , Speirs, D.C. , Hildrew, A.G. & Hal, C. (2005) Quantification and resolution of a complex, size‐structured food web. Advances in Ecological Research, 36, 85–135.

[ele14441-bib-0079] Zhang, H. , Väliranta, M. , Swindles, G.T. , Aquino‐López, M.A. , Mullan, D. , Tan, N. et al. (2022) Recent climate change has driven divergent hydrological shifts in high‐latitude peatlands. Nature Communications, 13(1), 4959. 10.1038/s41467-022-32711-4 PMC9402595 36002465

