# Supplementary material for: Long‐term data reveal widespread phenological change across major US estuarine food webs
Source: Ecol Lett. 2024 Dec 31;27(12):e14441. doi: 10.1111/ele.14441 (PMC11686945; doi:10.1111/ele.14441)
Supplement: Supplementary file 1 — Data S1. [file ELE-27-0-s001.docx]

**Long-term data reveal widespread phenological change across major U.S. estuarine food webs**

Robert J. Fournier, Denise D. Colombano, Robert J. Latour, Stephanie M. Carlson, Albert Ruhi

**Supplementary Materials**

***S1: Data quality and filtering criteria***

To ensure that individual taxa meet data density requirements for analyses, we implemented a multi-tiered filtering process. For inclusion in the final model, we required at least 10 years of data for each taxa (for the San Francisco Bay, age-0 and age-1+ fishes were analyzed separately) at each sampling location. For each taxa, we required at least 4 sampling visits that had non-zero catch data within a 7 month window per year. As sampling for fishes in the Chesapeake Bay only began in 2002, has a comparatively narrow sampling window, and the resulting dataset contains a much higher proportion of zero catch visits, we required at least 40 total visits at each site with non-zero catch for a given taxa over a minimum 10 year span for these taxa. For time series that fit these criteria, we then used autoregressive integrated moving average (ARIMA) models fitted with a Kalman expectation-maximization algorithm to interpolate missing abiotic covariate values (Comte et al. 2021). These models allowed for seasonality and deterministic drift within each time series. Because the data from each taxonomic group were taken from different monitoring programs at each estuary, the final data set contained time series representing 33 taxa in the San Francisco Bay (8 fishes, 19 zooplankton, 6 phytoplankton), 84 taxa in the Chesapeake Bay (5 fishes, 19 zooplankton, 60 phytoplankton), and 28 taxa in the Massachusetts Bay (14 zooplankton, 14 phytoplankton). For the San Francisco Bay, we accessed the pre-compiled fish and zooplankton data using the *Deltafish* (Clark and Bashevkin 2022) and *Zooper* (Bashevkin 2020) packages, respectively, in Program R (v 4.2.1; R Core Team 2022). From these time series, we identified the calendar day of peak abundance each year for each taxon at each sampling location and the corresponding mean annual temperature and salinity values. As sampling in Massachusetts Bay decreased from 12 months to 10 months per year in 2004, we calculated the mean annual temperature based only on months that had consistent sampling throughout the time series (February through October). Because abundance estimates can be influenced by sampling methodologies, we decided to use the date of peak abundance/biomass as our phenological response. This measure is agnostic of total abundance and facilitates comparability between taxa with disparate sampling. Because calendar day is a circular variable, there is a risk of peak abundance dates occurring towards the start and end of the year producing highly different results despite being temporally adjacent. To address this, we adjusted the calendar day within a year to a “species day” where the numbering of days starts 6 months prior to the mean peak abundance date. This way, most of the dates of peak abundance would occur within the middle of our constructed “year” –facilitating examination of linearized slopes.

*Data collection and synthesis*

Across all three systems, data for each trophic level were collected using various methodologies. Fishes in the San Francisco bay are collected via two separate trawl nets, an otter trawl (20.3 to 1.3 cm mesh) that targets benthic fishes and a midwater trawl that targets pelagic species (CDFW 2023a). We determined biomass for a given month at each sampling station by combining CPUE estimates for each of these nets. Planktonic taxa are sampled in three ways: macrozooplankton are sampled via a 505 µm mesh tow net, mezozooplankton are sampled via a 160 µm net, and microzooplankton are sampled via a teel pump with samples are passed through a 43 µm mesh filter (CDFW 2023b). Phytoplankton are assessed via a 60 mL water sample taken from a depth of 1 m at each sampling station. In the Chesapeake Bay, fishes are sampled via a bottom trawl with mesh ranging from 15.2 to 7.6 cm (Latour et al. 2023). Zooplankton and phytoplankton are sampled with a 500 µm tow net for macro and mezozooplankton; and a teel pump sample passed through a 44 µm mesh screen for microplankton (CBP 2023). In Massachusetts Bay, zooplankton are sampled via a 102 µm tow net, while phytoplankton are collected in 4 L water samples passed through a 20 µm mesh screen (MWRA 2023). To account for variability in methodologies, each estuary was modeled independently, and trivariate slopes were produced for each individual taxa. Thus, each taxon at each estuary was examined only using internally consistent data. Additionally, we included “sampling station” as a random effect in our models to account for a proportion of environmental and methodological heterogeneity at each sampling location. We also included the start and end years of each time series as random effects within the model.

**Table S1:** Data sources and monitoring program descriptions for data included in our models. *These programs started at 12 samples per year but decreased to 10 in 2004.

| **Taxa** | **Program** | **Years** | **Samples per year** | **Sampling locations** | **Gear types** | **Subregions** |
| --- | --- | --- | --- | --- | --- | --- |
| SFE Fishes | California Department of Fish and Wildlife’s San Francisco Bay Study | 1980-2020 | 12 | 39 | Graduated mesh otter trawl ranging from 20.5 to 1.3 cm  Graduated mesh midwater trawl ranging from 20.3 to 1.3 cm | Delta, Confluence, Suisun Bay, San Pablo Bay, Central Bay, South Bay |
| SFE Phytoplankton | California Department of Fish and Wildlife Environmental Monitoring Program | 2008-2021 | 12 | 8 | 60 ml water sample | Delta, Suisun Bay |
| SFE Zooplankton | California Department of Fish and Wildlife Environmental Monitoring Program | 1972-2021 | 12 | 54 | 505 µm tow net  160 µm tow net  Teel pump, 43 µm mesh net | Delta, Suisun Bay, San Pablo Bay, |
| CHE Fishes | Virginia Institute of Marine Science’s Chesapeake Multispecies Monitoring and Assessment Program | 2002-2022 | 9 | 44 | Graduated mesh trawl ranging from 15.2 to 7.6 cm | Upper Bay, Virginia Mainstem, Maryland Mainstem |
| CHE Zooplankton | Chesapeake Bay Program | 1985-2002 | 12 | 11 | 500 µm tow net  Teel pump, 44 µm zooplankton micro | Upper Bay, Virginia Mainstem, Maryland Mainstem |
| CHE Phytoplankton | Chesapeake Bay Program | 1985-2020 | 12 | 14 | 500 µm tow net  Teel pump,44 µm mesh | Upper Bay, Virginia Mainstem, Maryland Mainstem |
| MAS Zooplankton | Massachusetts Water Resources Authority’s Water Column Monitoring Program | 1992-2022 | 10* | 13 | 102 µm tow net | Massachusetts Bay, Cape Cod, Boston Harbor |
| MAS Phytoplankton | Massachusetts Water Resources Authority’s Water Column Monitoring Program | 1992-2022 | 10* | 13 | 4L water sample passed through 20 µm mesh screen | Massachusetts Bay, Cape Cod, Boston Harbor |

***S2: Environmental conditions at each estuary***


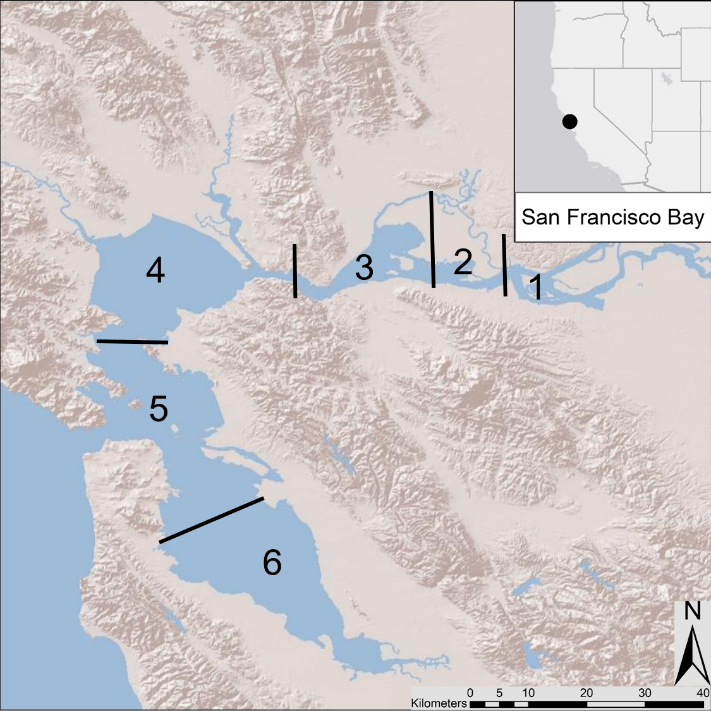


**Figure S1**: Map of San Francisco Bay with regional boundaries. Region 1 is the Delta, Region 2 is the Confluence, Region 3 is Suisun Bay, Region 4 is San Pablo Bay, Region 5 is the Central Bay, Region 6 is the South Bay.


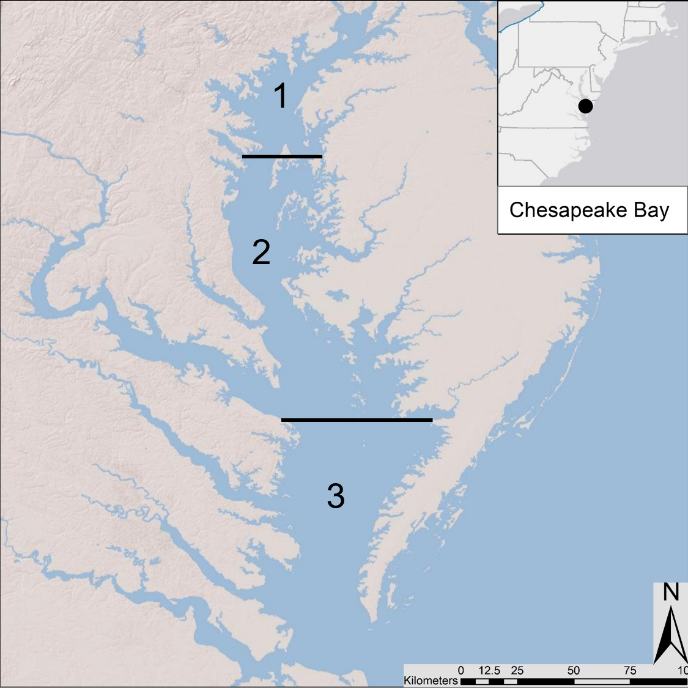


**Figure S2**: Map of Chesapeake Bay with regional boundaries. Region 1 is the Upper Bay, Region 2 is the Maryland Mainstem, Region 3 is the Virginia Mainstem.


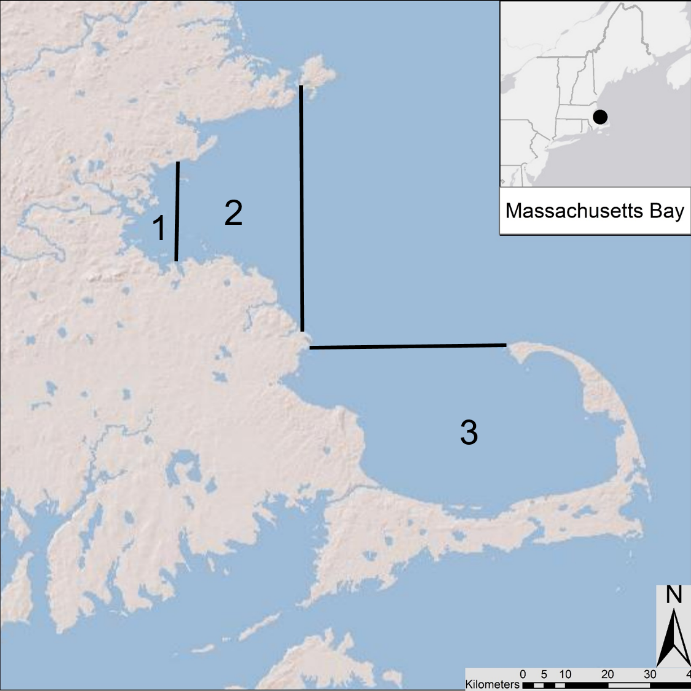


**Figure S3**: Map of Massachusetts Bay with regional boundaries. Region 1 is Boston Harbor, Region 2 is the main region of Massachusetts Bay, Region 3 is Cape Cod.

**Table S2:** Estuary subregions and sampling years.

| **Estuary** | **Region** | **Years** |
| --- | --- | --- |
| San Francisco | Delta | 1972-2021 |
| San Francisco | Confluence | 1972-2021 |
| San Francisco | Suisun Bay | 1972-2021 |
| San Francisco | San Pablo | 1980-2020 |
| San Francisco | Central | 1980-2020 |
| San Francisco | South | 1980-2020 |
| Chesapeake | Upper Bay | 1985-2022 |
| Chesapeake | Maryland Mainstem | 1985-2022 |
| Chesapeake | Virginia Mainstem | 1985-2022 |
| Massachusetts | Massachusetts Bay | 1992-2022 |
| Massachusetts | Cape Cod | 1992-2022 |
| Massachusetts | Boston Harbor | 1992-2022 |

**
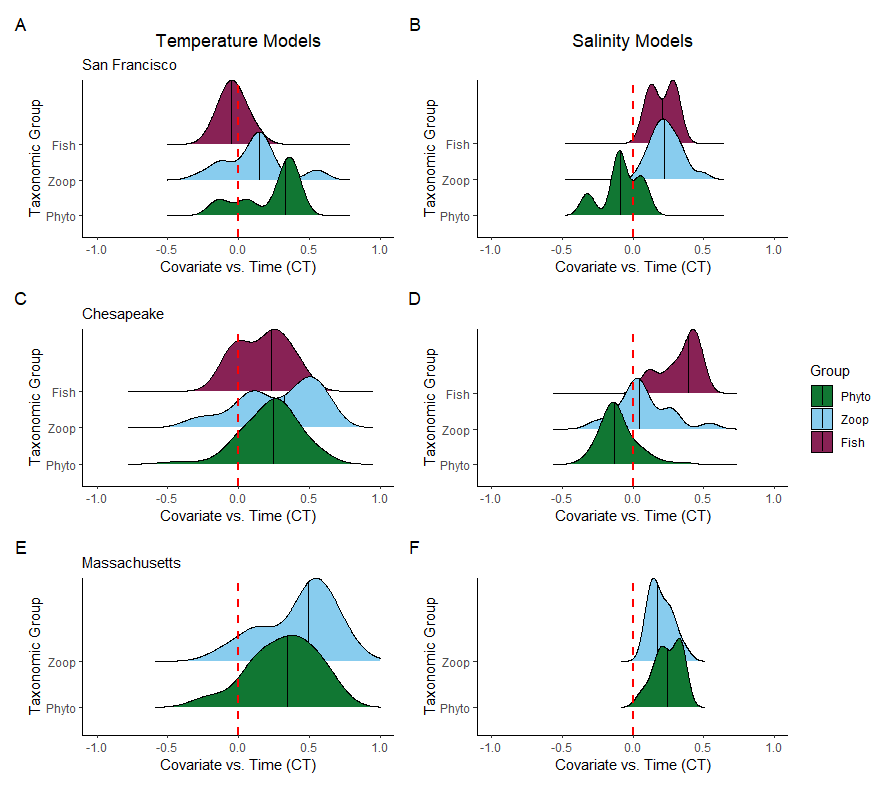
**

**Figure S4:** Density plots for modeled annual temperature (left) and salinity (right) slopes. The top row (A, B) represents the San Francisco Bay, the middle row (C, D) Chesapeake Bay, and the bottom row (E, F) Massachusetts Bay.


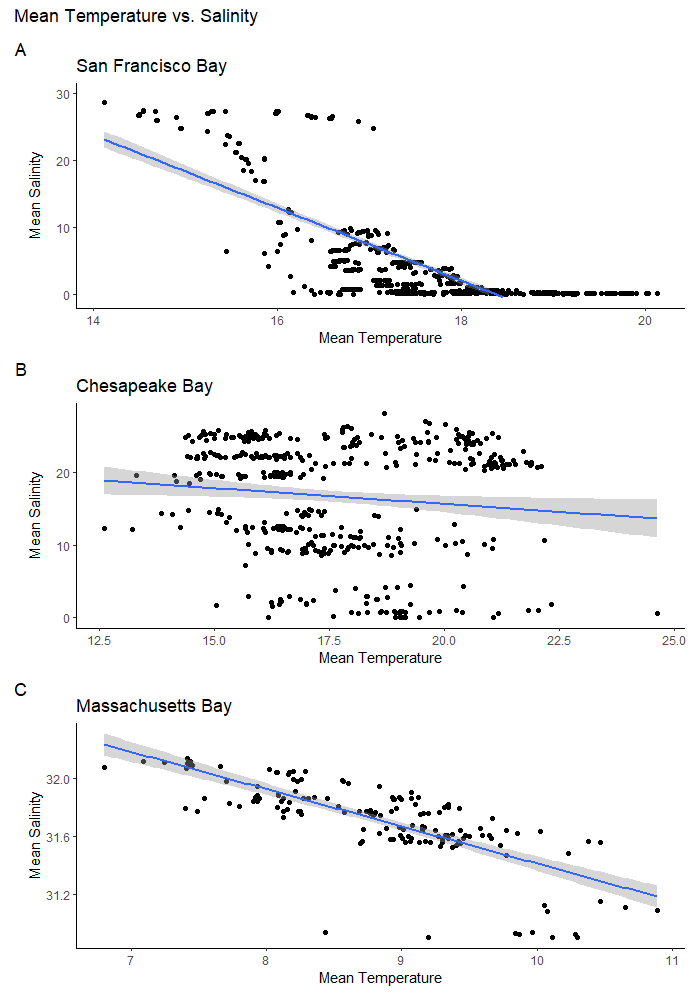


**Figure S5**: Association plot between mean temperature (X-Axis) and Mean Salinity (Y-axis) for the San Francisco (A), Chesapeake (B) and Massachusetts (C) Bays.

***S3: Trivariate metaregression model***


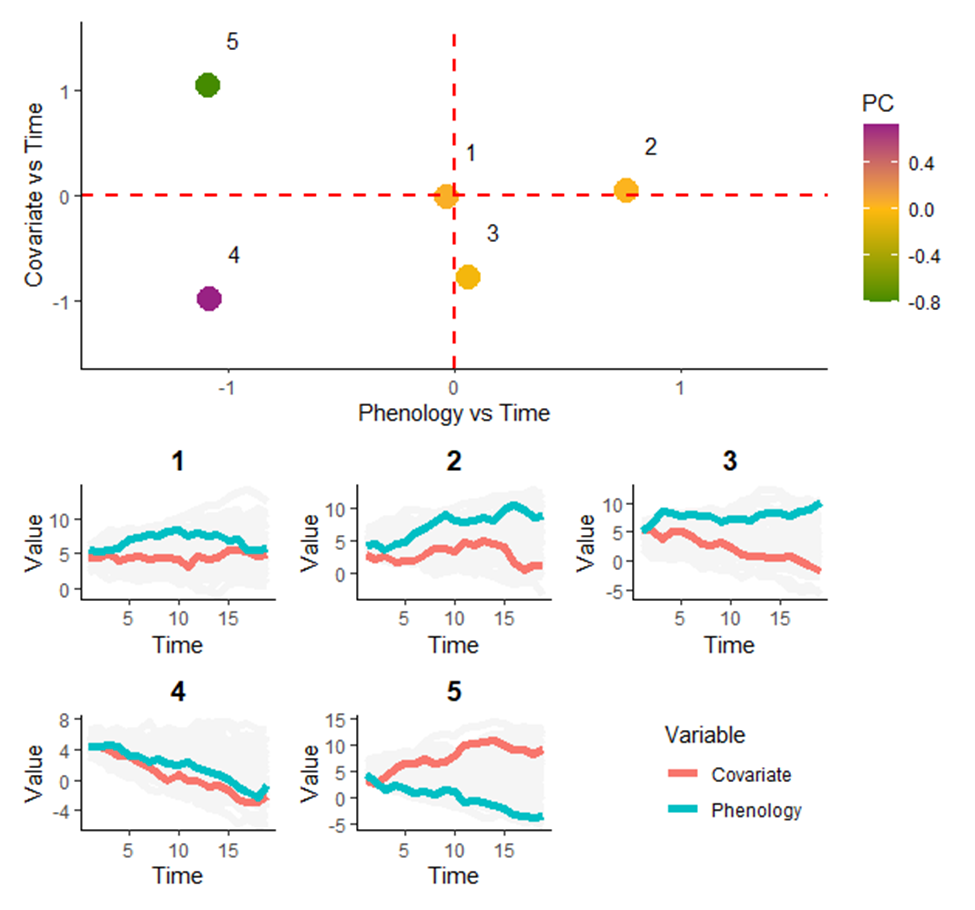


**Figure S6:** A) Conceptual diagram for the results of trivariate mixed effects models. Relationships between phenology (calendar day of peak abundance) and time (year) are on the X axis. Values less than 0 indicate advancing (earlier) phenologies, while values greater than 0 indicate delaying phenologies. The relationship between the covariate (temperature or salinity) and time is on the Y axis. Values less than zero indicate a negative trend, while values greater than zero indicate a positive trend. The relationship between the phenology and the covariate is indicated by color. In particular, values less than 0 (green) indicate that a higher covariate advances phenology (while lower values delay). Values greater than 0 (purple) indicate that a higher covariate delays phenology (while lower values advance). Each point is the result of 1000 simulations for both a phenology time series and a covariate time series. Each time series was built with a deterministic drift value (either -0.2, 0, or 0.2) to influence trends in the desired direction. Additionally, each simulation had a process error variance of 0.5.
B) Examples of simulated time series included in our model. For point 1, both phenology and covariate time had no associated trend. For point 2, the phenology had a positive trend, but the covariate had no trend. For point 3, the phenology had no trend, but the covariate had a negative trend. For point 4, both the phenology and covariates had negative trends. For point 5, the covariate had a positive trend while the phenology had a negative trend.

**
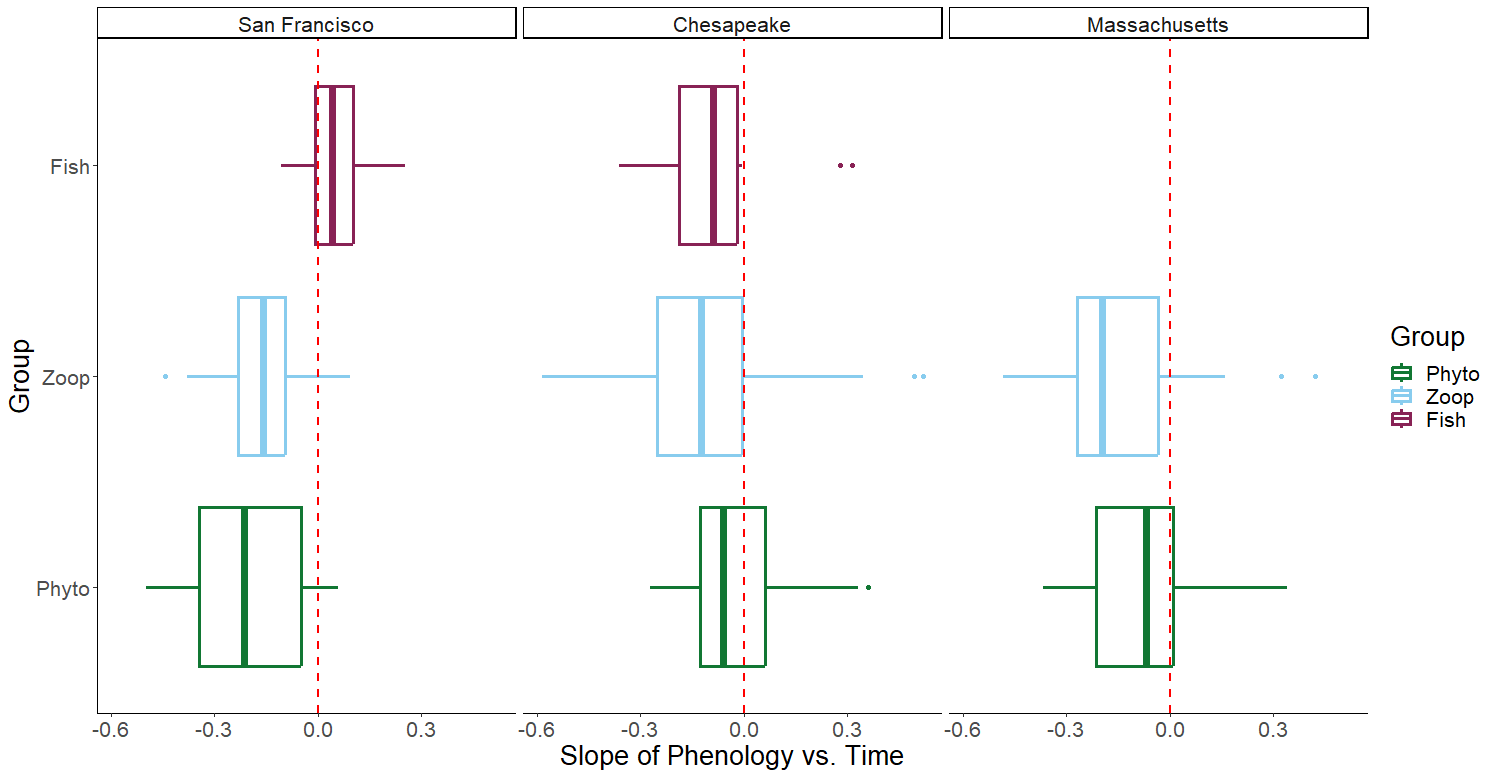
**

**Figure S7:** Distribution of phenology vs time (*PT*) slopes at each estuary. Values to the left of zero indicate advancing phenologies, values to the right of zero indicate delaying phenologies.

**
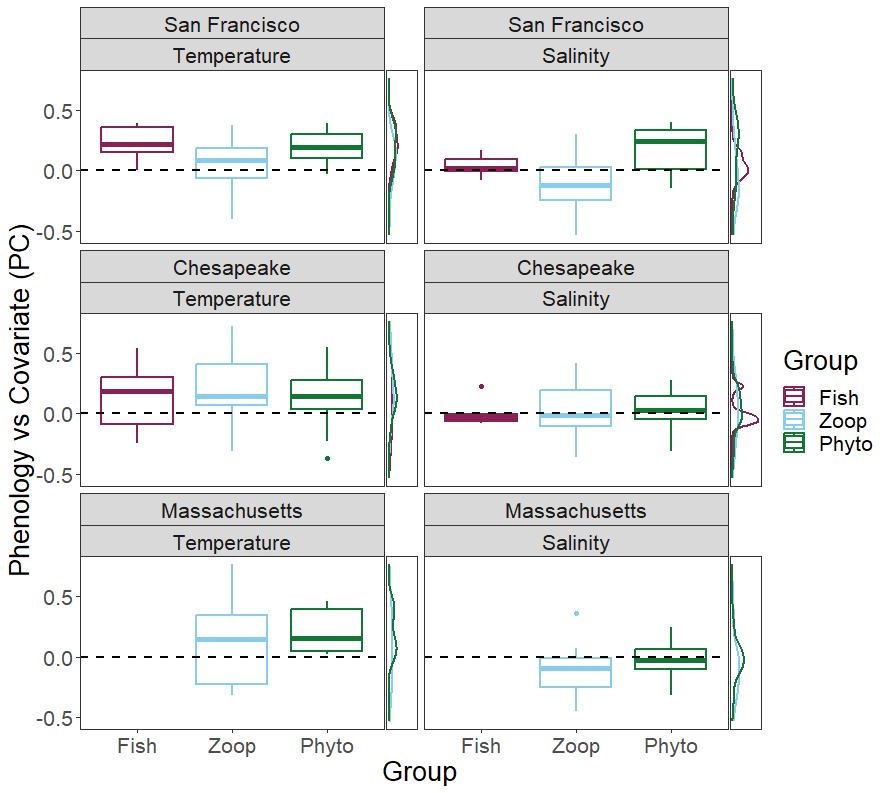
**

**Figure S8:** **Associations between phenology and climate covariates, across trophic levels and estuaries.** All estimated phenology vs. covariate slopes (*PC*), obtained from temperature (left column) and salinity models (right column), are shown for each estuary: San Francisco Bay (first row), Chesapeake Bay (second row), and Massachusetts Bay (third row). Side plots represent the density of slope values across trophic levels. *PC* slopes represent the magnitude and direction of sensitivity of phenological change to climate change.

***S4: Temporal Autocorrelation and Model Validation***

Because our models incorporate data repeatedly sampled through time, but do not explicitly account for temporal autocorrelation, we sought to examine what effects any potential autocorrelation might have on phenological and environmental time series and subsequent model results. First, we calculated the autocorrelation function (ACF) for each time series, and examined autocorrelation coefficients of up to 5 lags (i.e., 5 steps, or years, ahead). This window is most likely to show significant autocorrelation, if it exists; even if annual data are less likely to show autocorrelation relative to monthly or daily data (Tu et al. 2023). We found that autocorrelation of phenology values varied across lags and systems (Figure S9), however, the median distribution of ACF values was close to zero, and the vast majority of the time series (90-98.5% of them) had no significant ACF at lag 1. We saw similar results for environmental time series, with only 10-28% of time series displaying significant autocorrelation at lag 1 for temperature, and 0-15% for salinity (Figure S10)


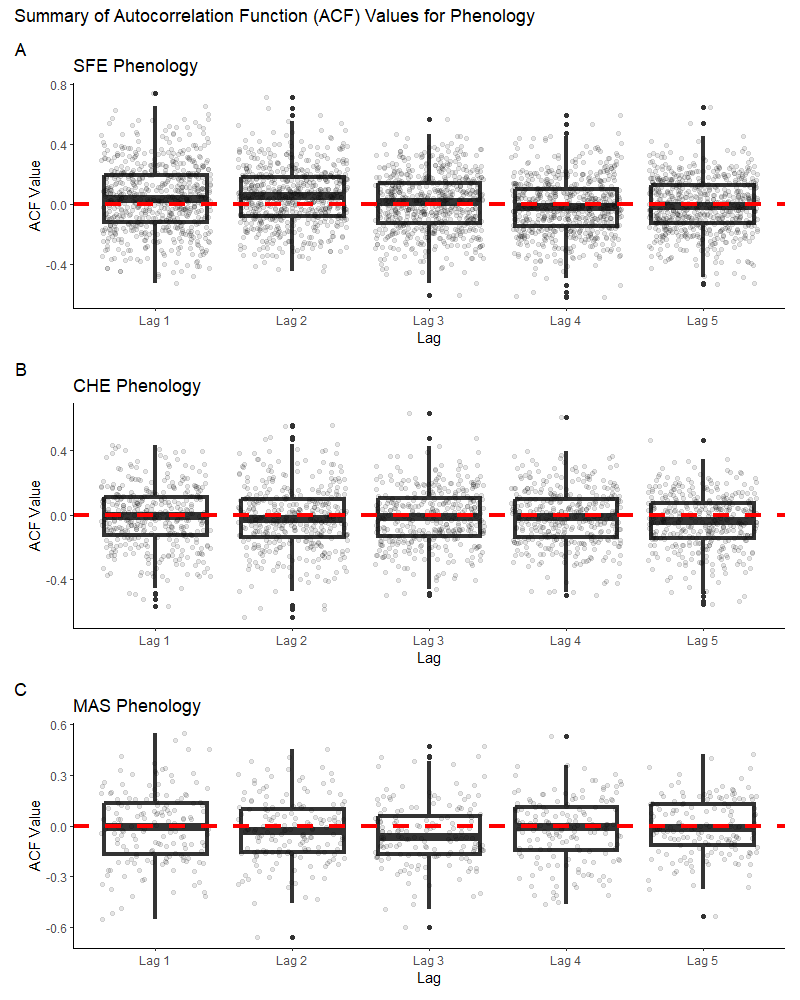


**Figure S9**: Autocorrelation function (ACF) values for each phenological time series in San Francisco Bay (A), Chesapeake Bay (B) and Massachusetts Bay (C). Each lag represents one year (i.e., lag-1 autocorrelation captures the autocorrelation, or ‘memory’, of a year’s phenology on next year’s phenology).


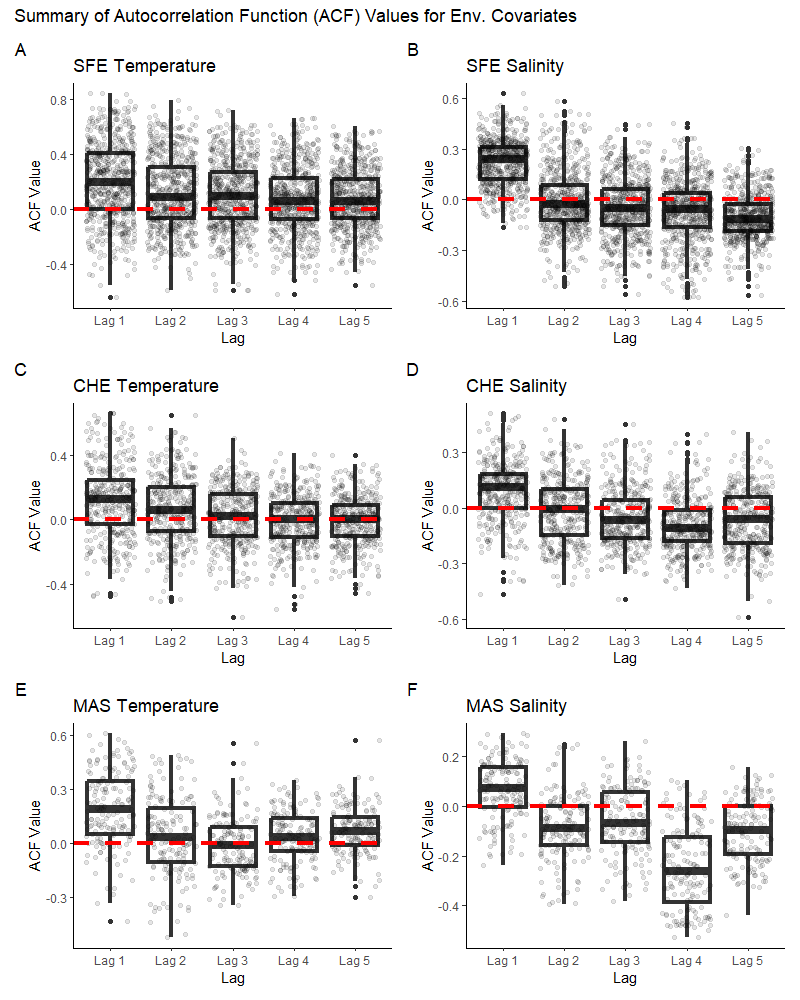


**Figure S10**: Autocorrelation function (ACF) values for each environmental time series in San Francisco Bay (A, B), Chesapeake Bay (C, D) and Massachusetts Bay (E, F). Each lag represents one year. (i.e., lag-1 autocorrelation captures the autocorrelation, or ‘memory’, of a year’s environmental conditions on next year’s conditions).

We then explored whether systematic variation in temporal autocorrelation could be influencing our findings. To this end, we first compared simple linear regression slopes from the phenological (i.e., the date of peak abundance for each year) and environmental time series for each taxa at each sampling station to those obtained from a Theil-Sen test. Thiel-Sen is a median-based regression model that ‘breaks’ autocorrelation by comparing all pairs of points and taking the median value to calculate a regression slope (See Figure S11). We found that simple linear slopes very closely tracked Theil-Sen slopes, following associations close to 1:1 (b=0.81-1.1). This result supports that variation in phenological and environmental trends observed via simple linear regression is not driven by systematic variation in temporal autocorrelation–in other words, temporal autocorrelation is not driving long-term trends.


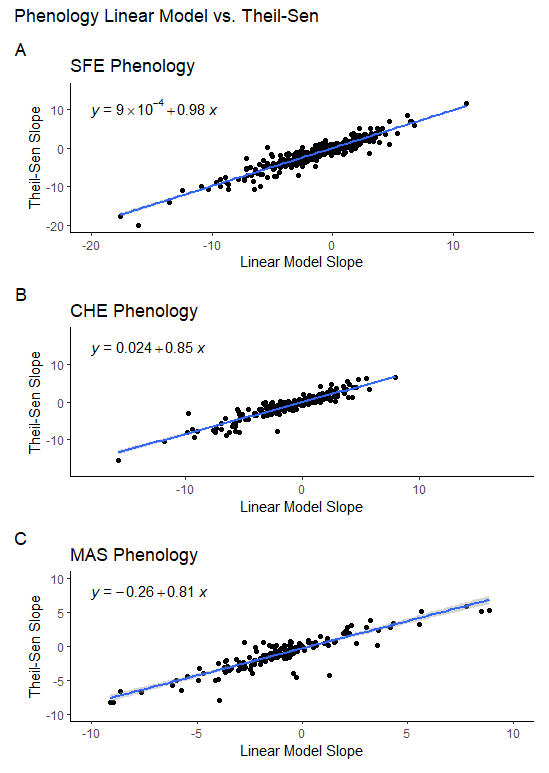


**Figure S11**: Comparison of phenological trends obtained via simple linear regression (X) versus Theil-Sen robust estimator (Y), for the San Francisco (A), Chesapeake (B), and Massachusetts (C) Bays. The equation for a simple linear regression is provided.


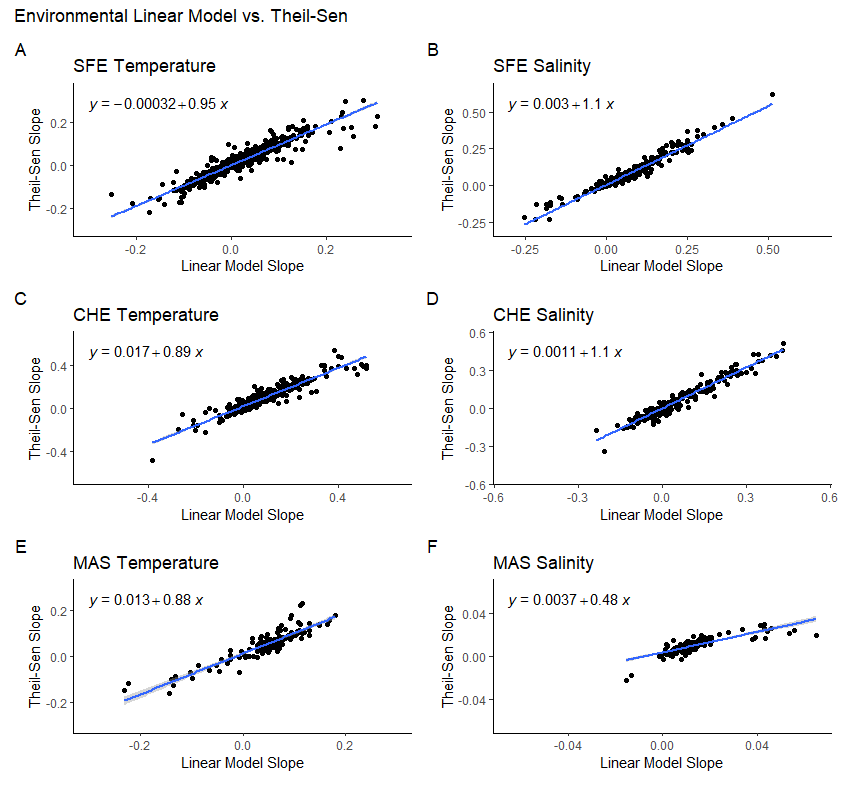


**Figure S12**: Climatic trends (temperature or salinity over time) obtained via simple linear regression (X) versus the Theil-Sen robust estimator (Y), for San Francisco Bay (A, B), Chesapeake Bay (C, D) and Massachusetts Bay (E, F). Temperature values are in the left column (A, C, E), while salinity values are on the right (B, D, F).

Next, as our model inputs are transformed effect sizes calculated from Pearson correlation coefficients, we wanted to see if similar patterns held when looking at associations. So, we compared the Pearson correlation coefficients to coefficients calculated by Kendall’s Tau Rank Correlation Coefficient. Like Theil-Sen slopes, Kendall’s Tau disrupts temporal autocorrelation by ranking the data within each of the quantiles and measuring the concordance (or discordance) between any given pair of points. Once again, we found strong agreement between Pearson coefficients and Kendall’s Tau (Figures S13, S14).


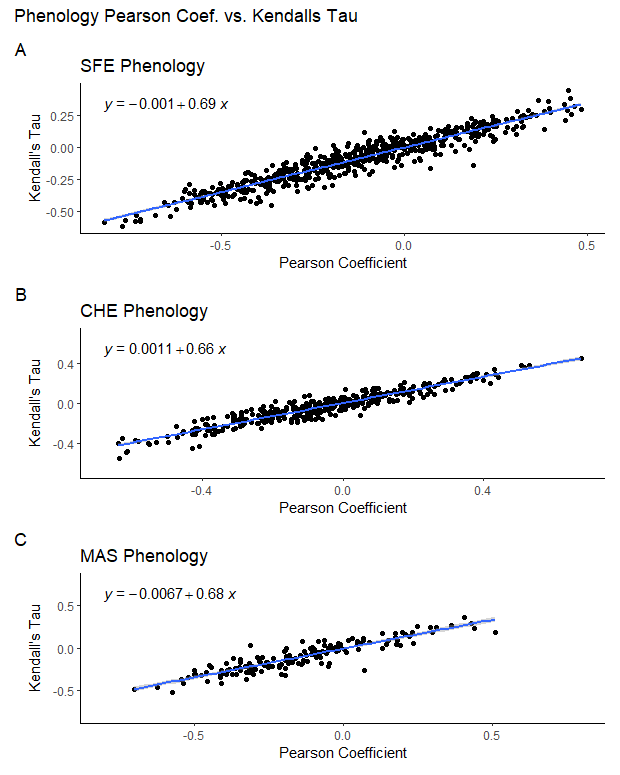


**Figure S13**: Phenological trends (phenology over time) obtained via Pearson correlation coefficients (X) versus Kendall’s Tau coefficients (Y) for the San Francisco (A), Chesapeake (B), and Massachusetts (C) Bays.


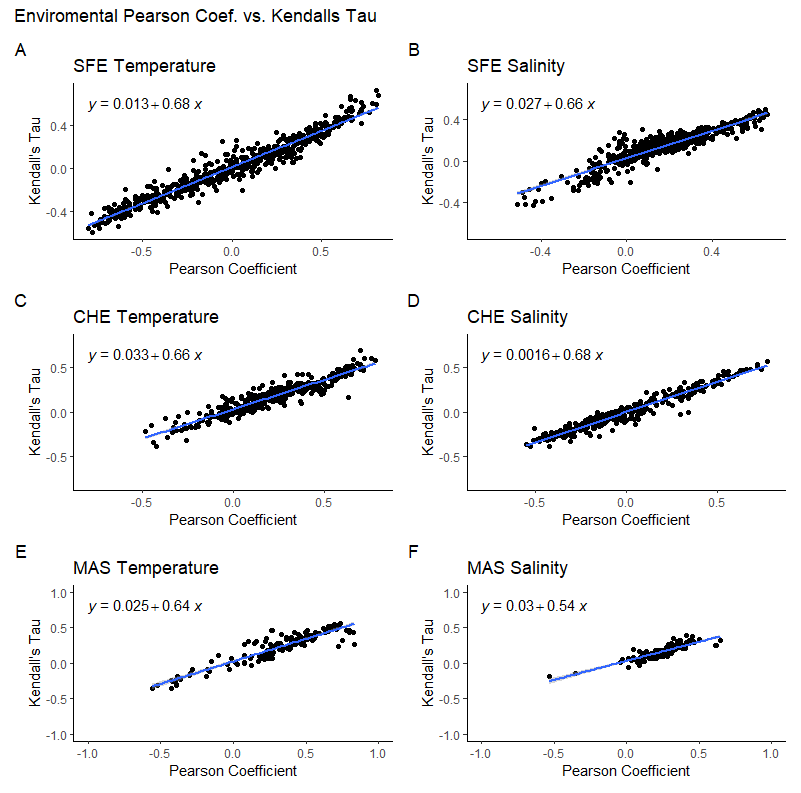


**Figure S14**: Climatic trends (temperature or salinity over time) obtained via Pearson correlation coefficients (X) versus Kendall’s Tau coefficients (Y) for San Francisco Bay (A,B), Chesapeake Bay (C, D) and Massachusetts Bay (E, F). Temperature values are in the left column (A, C, E), while salinity values are on the right (B, D, F).

Finally, we sought to explore if linear trends in the environmental covariates could be biased by other sources of variation (i.e. process error or ‘noise’). This gets at the question of whether strong environmental changes could be influenced by noise. To do this, we fitted autoregressive (AR) order 1 models to environmental time series (Hampton et al 2013). These models explicitly remove autocorrelation, and provide estimates of directional change through time (U) and process error variance (Q), the latter capturing environmental cycles (periodicity) and ‘excursions’ (stochastic variation) in the data. We found that directional trends (U) were generally not associated with process error variance (Q). This result means that even if there is variation in the environmental data that is not captured by the long-term, directional trend (U), periodic and stochastic variation (captured in Q) are not biasing our inferences about long-term trends (Figure S15).


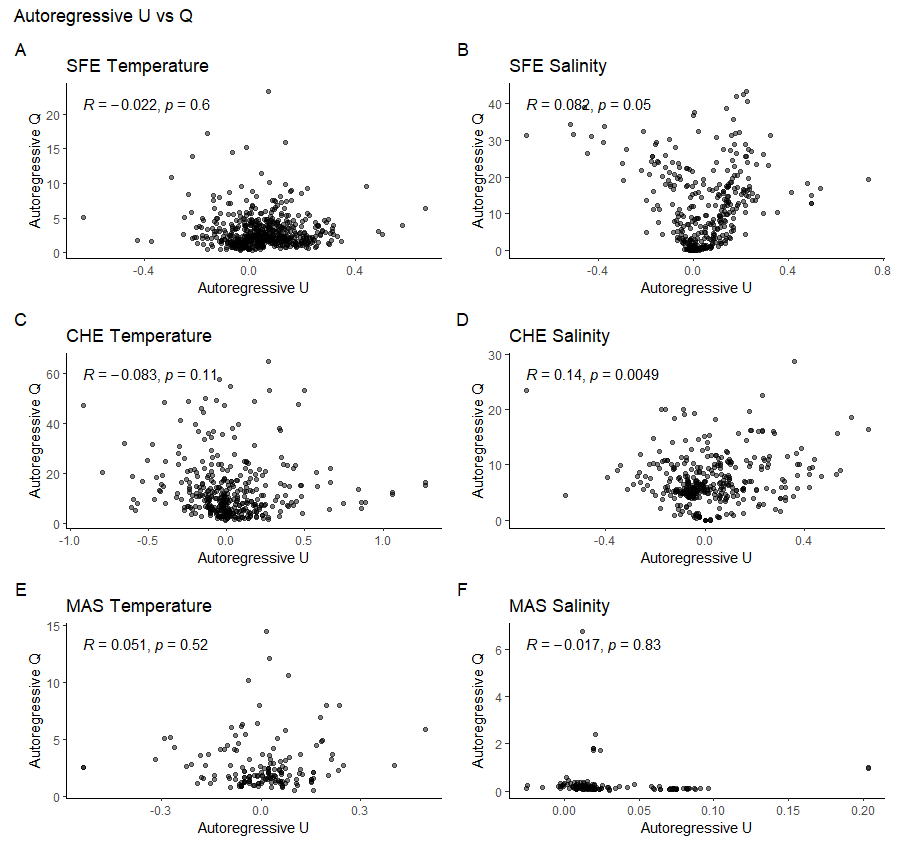


**Figure S15**: Rate of deterministic change (U) from an AR 1 model (X-axis) versus estimated process error variance (Q, Y-axis) for San Francisco Bay (A,B), Chesapeake Bay (C, D) and Massachusetts Bay (E, F). Temperature values are in the left column (A, C, E), while salinity values are on the right (B, D, F).

Collectively, these analyses indicate that our models were robust to the effects of temporal autocorrelation in the environmental data, as well as to the effects of non-linear variation in the data. However, we acknowledge that the inability to explicitly account for autocorrelation with our model structure is a potential limitation of our approach.

***S5: Comparison between annual and seasonal scales***

We also fitted models that incorporate seasonal climate data (i.e., temperature or salinity at the peak, rather than annual means). When we explored climatic sensitivity at this scale, we found that phenology-climate relationships (*PC*) of our modeled taxa differed by covariate (temperature or salinity, F_1,49.906_, p<0.001) system (F_2,10.397_, p<0.001), and by taxonomic group (F_2,37.671_, p<0.001), as well as the interaction between covariate and system (F_2,26.232_, p<0.001). Additionally, we found that taxa differentially tracked seasonal salinity trends in the San Francisco and Chesapeake bays (SFE seasonal X^2^_2,42.023,_ p<0.001, CHE seasonal X^2^_2,42.058,_ p<0.001) and temperature in the Chesapeake (CHE X^2^_2,7.9646,_ p=0.018). We also compared rates of tracking between seasonal and annual scale climatic trends. In San Francisco Bay, 51.1% of taxa tracked annual temperature trends, while 44% tracked annual salinity trends. However, a much higher proportion of taxa tracked seasonal trends in temperature (81.1%) than tracked salinity (33.3%). In Chesapeake Bay, 46.4% of taxa tracked annual temperature trends while 65.47% of taxa tracked seasonal temperature trends. Meanwhile, 71.42% of taxa annual salinity trends while 79.76% track seasonal salinity trends. Finally, 32.1% of taxa in Massachusetts Bay tracked annual temperature trends compared to 37.5% that tracked seasonal trends while the number of taxa that tracked annual and seasonal salinity trends are equal (57.1%). In general, most of the taxa that tracked both annual and seasonal models were categorized as not shifting phenologies within a stable environment. However, among shifting taxa, differences in tracking rates between annual and seasonal scales were largely driven by plankton species, which are much more likely to track seasonal than annual covariate trends. (Fig. 4, Supplementary Figure S18).

**
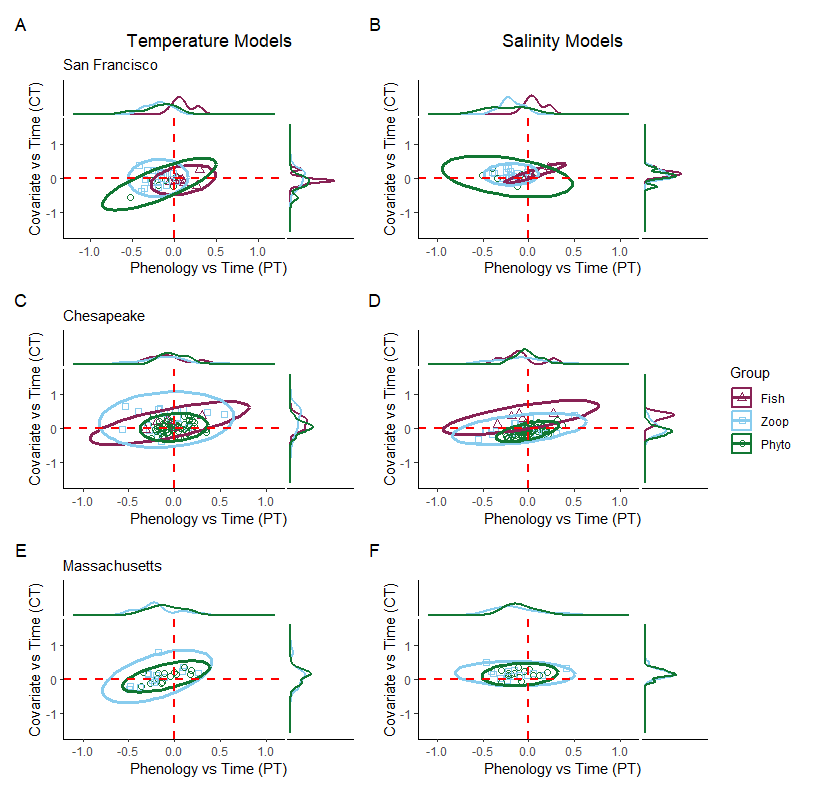
**

**Figure S16:** Diversity of phenological shifts and local climate trends, across trophic levels and estuaries for seasonal models. Estimated phenology vs. time slopes (*PT*, x axes, values above zero indicate delaying phenologies, values below zero indicate advancing phenologies) and local climate trend slopes (*CT*, y axes, values above zero indicates increasing temperature or salinity, below zero indicates decreasing temperature or salinity) are shown for each estuary. Left column: slopes from models estimating temperature effects; right column: slopes from models estimating salinity effects. For example, a taxon in the top left quadrant of panel A would be advancing its phenology in a warming climate (or in the bottom left quadrant, it would be advancing its phenology in a cooling climate). Similarly, a taxon in the top left quadrant of panel B would be advancing its phenology in an estuary that is increasing in salinity (or in the bottom left quadrant, it would be advancing its phenology in an estuary that is decreasing in salinity). For each estuary and model type, Bayesian 95% confidence ellipses were fitted to visualize the diversity of phenological and local climatic trends experienced by each taxonomic group (Fish, Phytoplankton, Zooplankton).

**
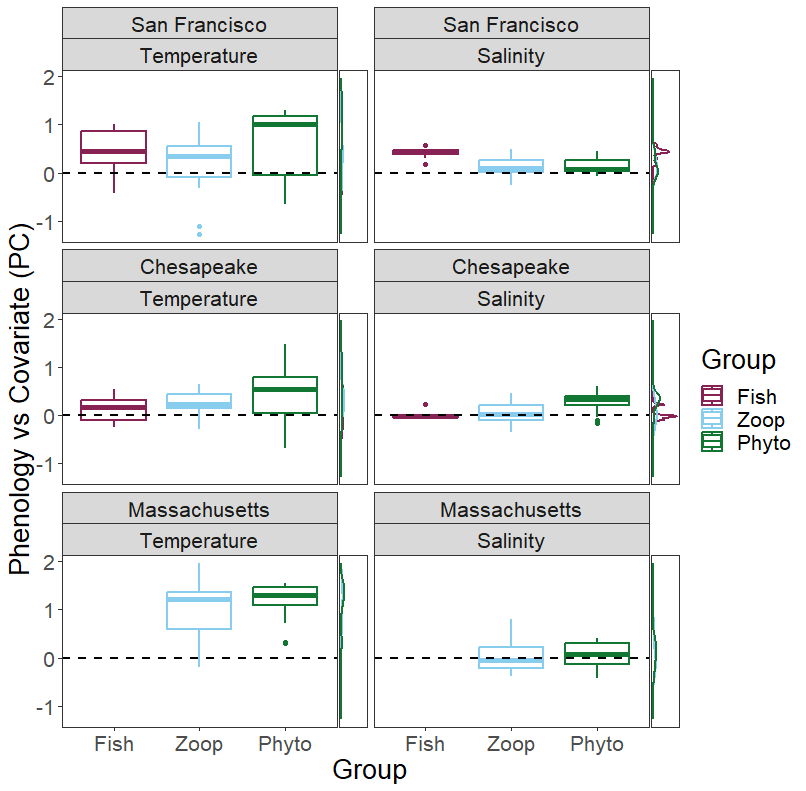
**

**Figure S17:** Associations between phenology and climate covariates, across trophic levels and estuaries for seasonal models**.** All estimated phenology vs. covariate slopes (*PC*), obtained from temperature (left column) and salinity models (right column), are shown for each estuary: San Francisco Bay (first row), Chesapeake Bay (second row), and Massachusetts Bay (third row). Side plots represent the density of slope values across trophic levels. *PC* slopes represent the magnitude and direction of sensitivity of phenological change to climate change.

**
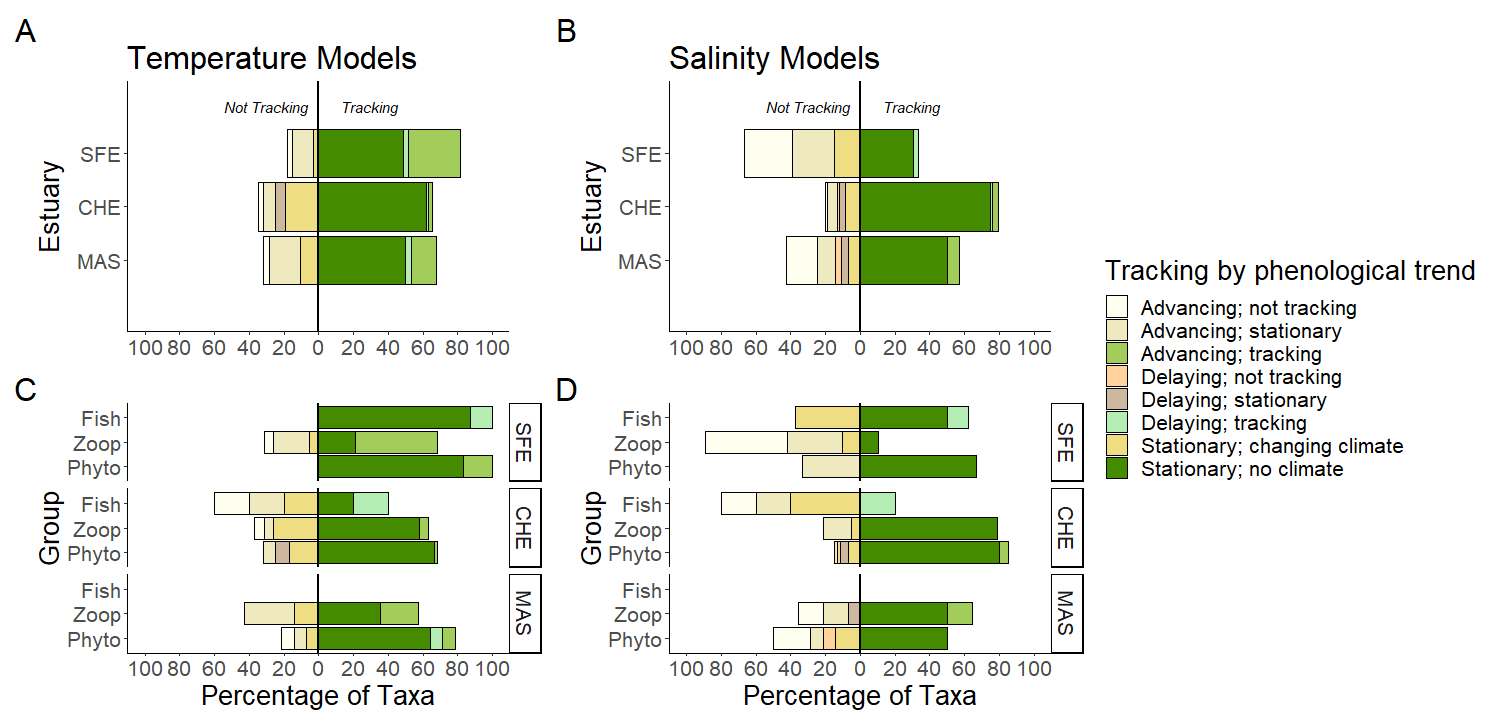
**

**Figure S18:** Left column: Proportion of all modeled taxa at each estuary tracking *vs.* not-tracking seasonal temperature (A, C) and seasonal salinity trends (B, D). For all panels, bars growing towards the left of zero represent taxa not tracking climate trends; bars growing towards the right of 0 represent taxa tracking climate trends. Additionally, shifts are noted as advancing or delaying (see color legend). We considered taxa track their climate those that 1) shifted their phenology, 2) existed within a non-stationary local climate, and 3) had a significant *PC* slope that was consistent with climatic and phenological trends (e.g., a positive *PC* slope when the taxa is advancing its phenology with a negative *CT* slope). Additionally, organisms that were not shifting their phenologies were considered to be tracking stationary climates. Organisms not tracking climate are broken down into non-shifting taxa in changing climates (“Stationary, no climate”), shifting taxa within stationary climates (see color legend, ‘“Not tracking (stationary)”), or taxa without a significant *PC* slope and/or a *PC* slope not consistent with climatic and phenological trends (“not tracking”).

**
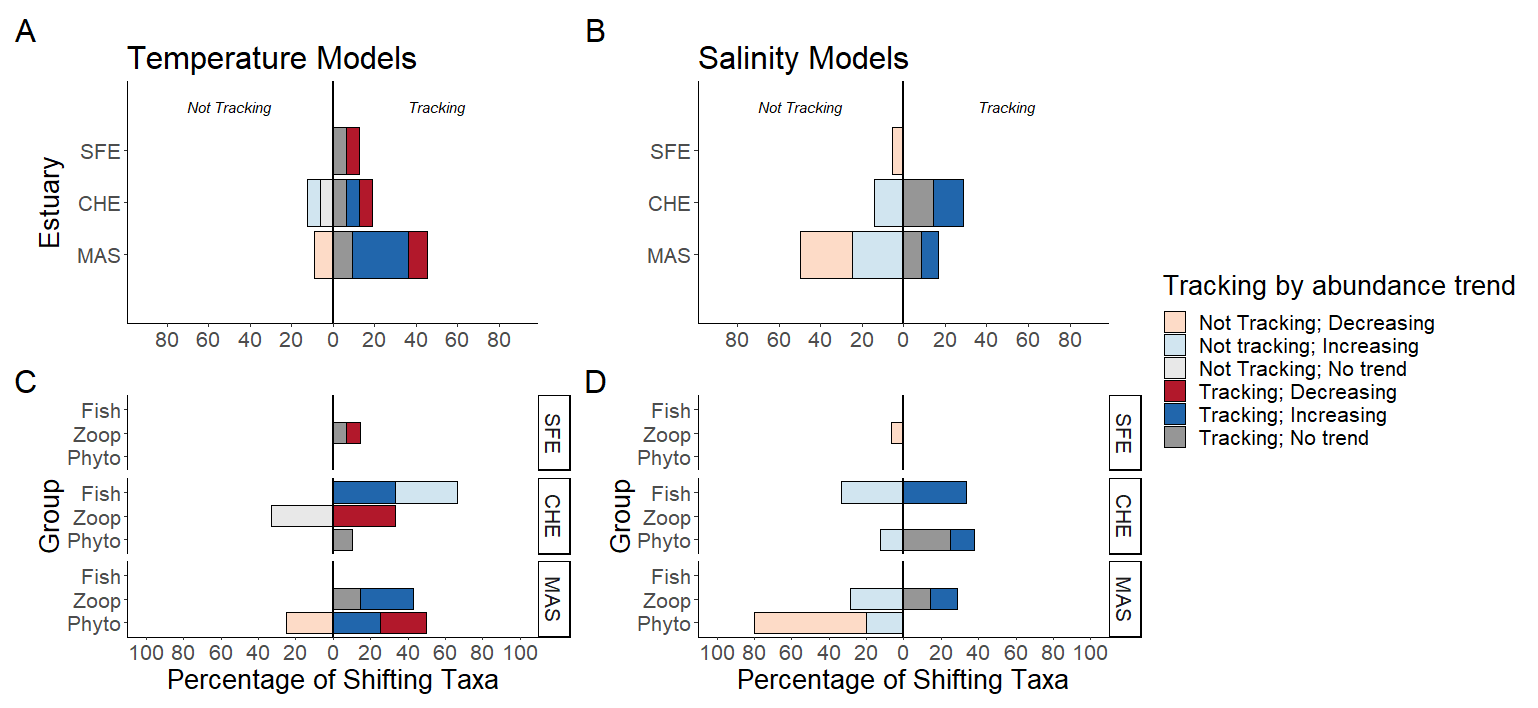
**

**Figure S19:** Right column: Abundance trends of taxa that are shifting phenology with respect to changing seasonal temperature (A, C) and seasonal salinity trends (B, D). For all panels, bars growing towards the left of zero represent taxa not tracking climate trends; bars growing towards the right of 0 represent taxa tracking climate trends. We considered taxa track their climate those that 1) shifted their phenology, 2) existed within a non-stationary local climate, and 3) had a significant *PC* slope that was consistent with climatic and phenological trends (e.g., a positive *PC* slope when the taxa is advancing its phenology with a negative *CT* slope).

***S6: Additional food web models***

We presented the results from regional food web models for San Francisco in the main text as the data encompassed all three trophic levels until the present. However, we also constructed regional models for Chesapeake and Massachusetts bays. In Chesapeake Bay, we found 7 advancing trends among phytoplankton taxa, and 2 advancing fish taxa. We also found that 4 phytoplankton and 1 fish taxa were delaying phenologies. Shifts were most common in the Maryland mainstem region (n=9). This region is also an intermediate salinity zone bookended by the fresher Upper Bay and the saltier Virginia mainstem (Supplementary figures S9, S10). We found no significant divergence in *PT* trends between groups in the Maryland mainstem (F_1,7.129_, p=0.009), Virginia mainstem (F_1,0.121_, p=0.72), or the Upper Bay (F_1,1.829_, p=0.18). We observed 12 shifts in Massachusetts Bay (11 advancing and 1 delaying). The main region of Massachusetts Bay had a higher number (n=9) of shifts than Cape Cod (n=3). These patterns resulted in significant differences in *PT* slopes between groups in Mass. Bay (F_1,10.62_, p=0.002), but not Cape Cod (F_1,0.926_, p=0.349; Supplementary figure S11).


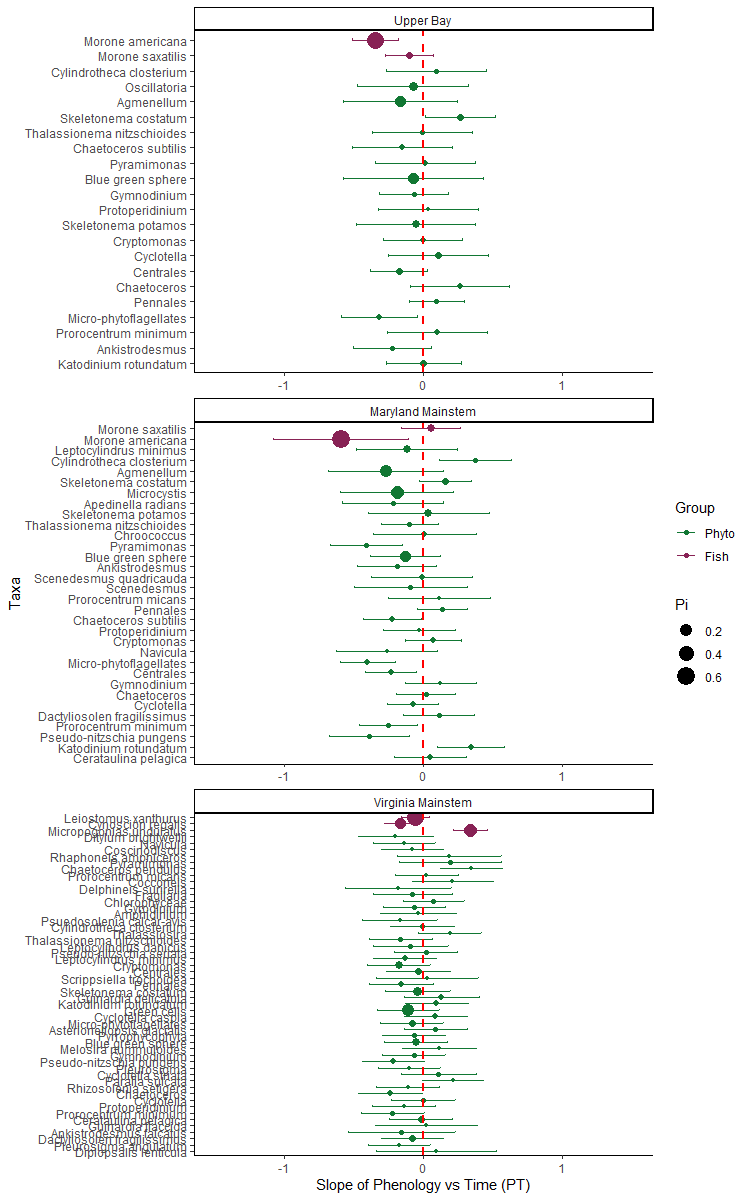


**Figure S20 (previous page)**: Food web models for the Chesapeake Bay. Individual points represent the maximum likelihood estimate for the slope and associated 95% confidence intervals. Points and CI’s to the left of zero are advancing their phenologies, while points and CI’s to the right of the zero are delaying their phenologies (color coded by trophic level, see legend). Point size represents relative mean abundance within the relevant trophic level.


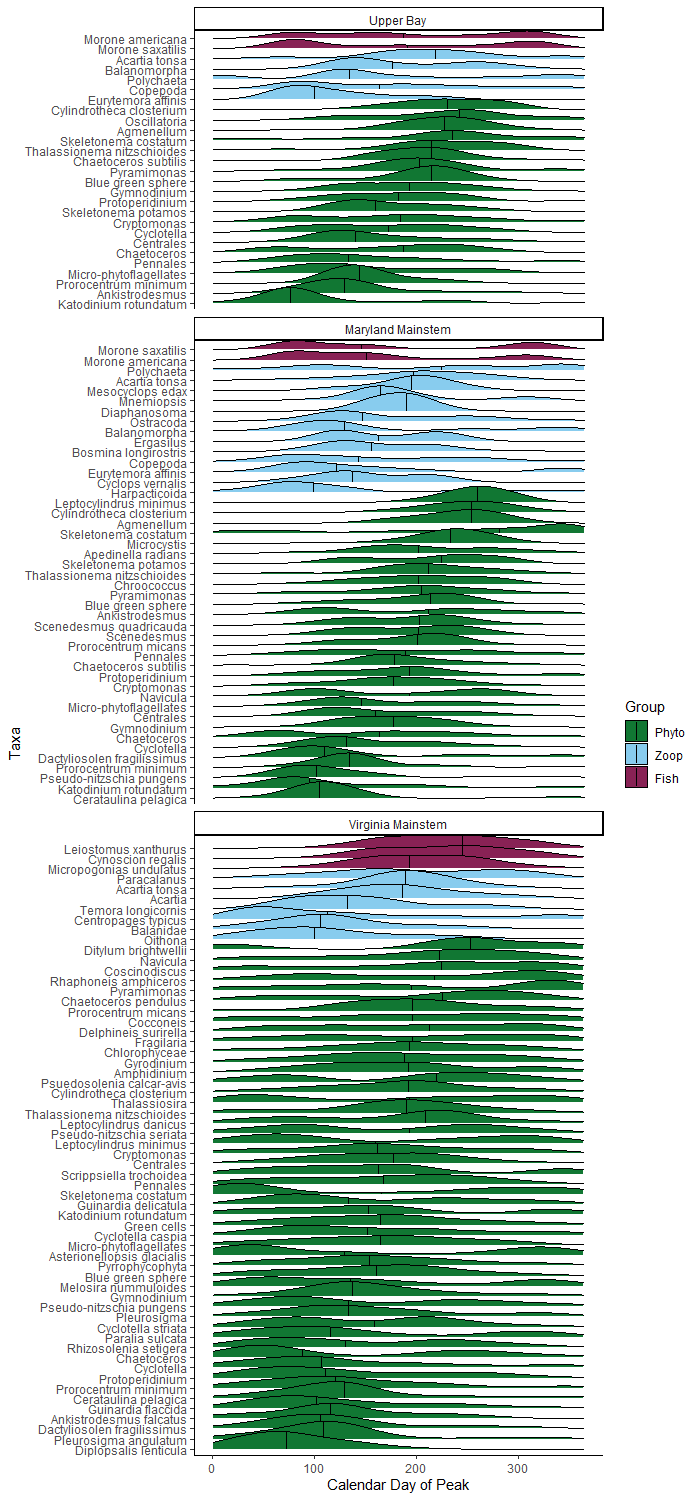


**Figure S21**: Distribution of the calendar day of peak abundance for taxa in each regional food web within the Chesapeake Bay.


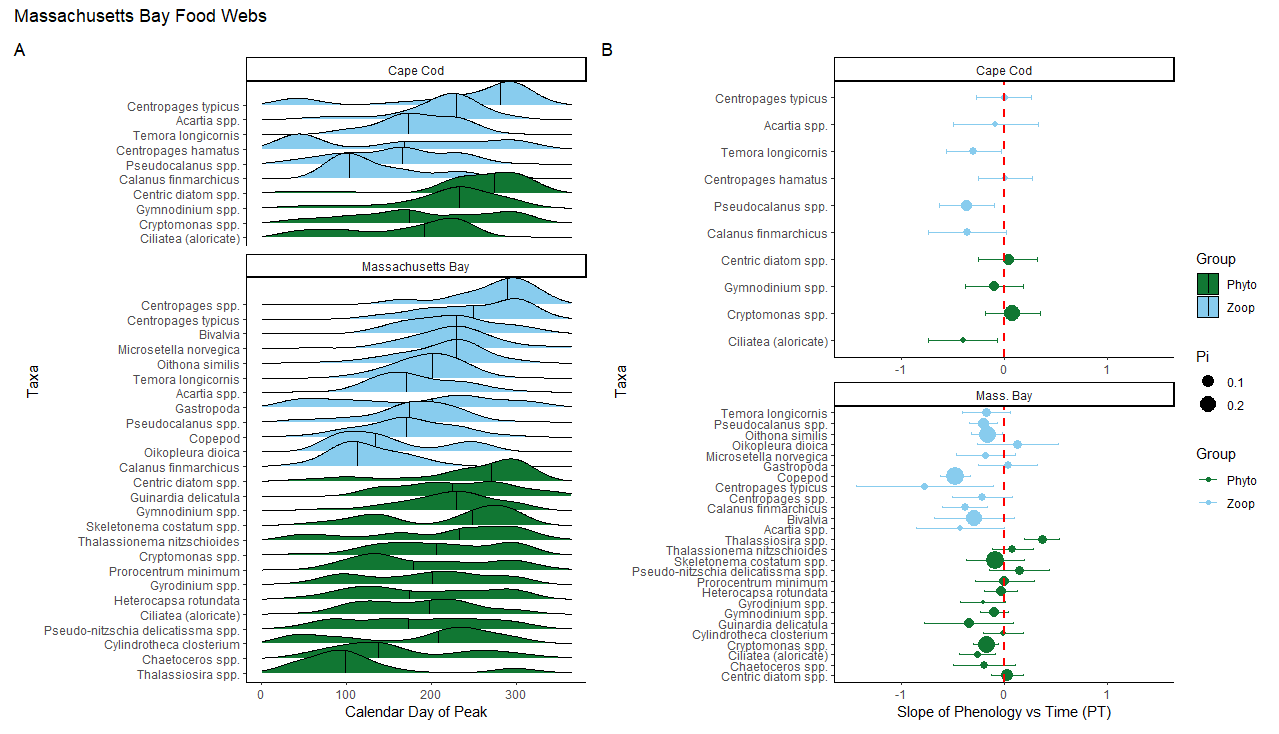


**Figure S22**: Food web models for Massachusetts Bay. A) Distribution of the calendar day of peak abundance for taxa in each regional food web within the Chesapeake Bay. B) Individual points represent the maximum likelihood estimate for the slope and associated 95% confidence intervals. Points and CI’s to the left of zero are advancing their phenologies, while points and CI’s to the right of the zero are delaying their phenologies (color coded by trophic level, see legend). Point size represents relative mean abundance within the relevant trophic level.

Throughout the main text and supplementary, we provide phenological results for all modeled species within a given region. However, temporal and spatial overlap does not necessarily translate to interaction. While we did not directly compare individual sets of interacting predator and prey species, we sought to provide a potential application of our food web models by exploring the phenology of a fish of conservation concern, the longfin smelt (*Spirinchus thaleicthys*), and several of its known prey items in San Francisco Bay (via Lojkovic Burris et al. 2022).

When we subsetted our results to only the Longfin smelt and its known prey in regions where they co-occur, we found that Longfin smelt tended to display historical peaks that overlapped, at least to an extent, with the historical peaks of several of its known prey items. Furthermore, we found that phenological trends seem to be diverging, with the predator peaking later in the year while most of its prey peak earlier. We know that Longfin smelt are often generalists, and will consume each of the prey taxa present in the subset. Therefore, if predator and prey do diverge in time, the degree of reliance on each prey item might change. We encourage continued exploration of patterns that more explicitly link predator and prey.


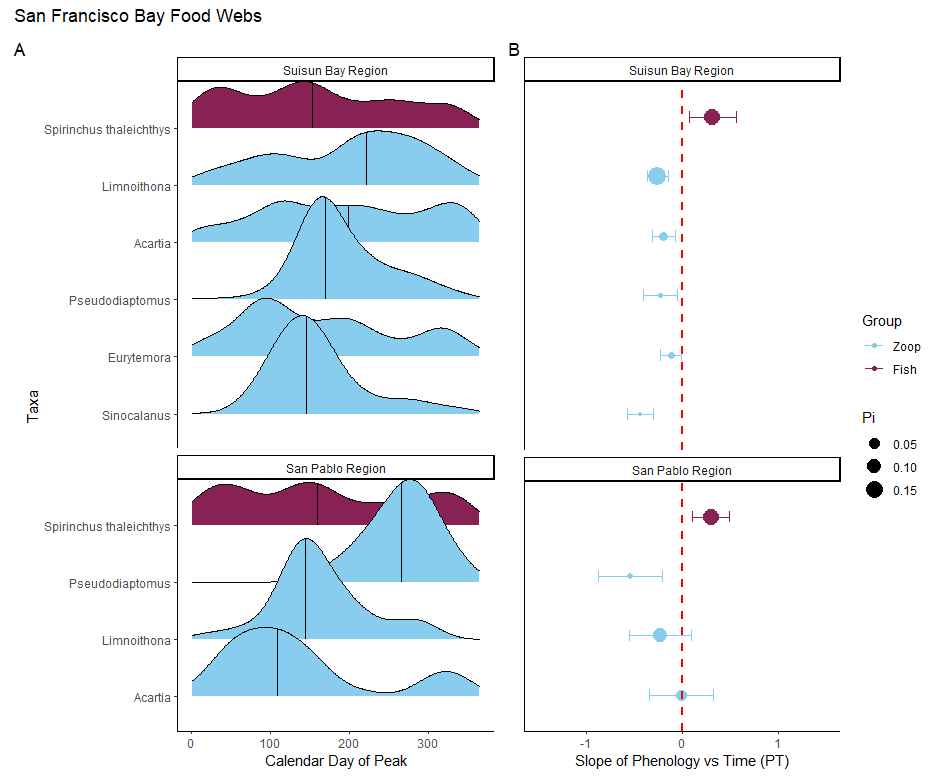


**Figure S23**: A) Distribution of historical dates of peak abundance for Longfin smelt (purple) and zooplankton (blue) in Suisun and San Pablo Bays. B) Modeled phenology vs. time slopes. Points to the right of the line are delaying phenology while points to the left are advancing. Points are sized according to relative abundance within the region.

***Supplemental References:***

Bashevkin, S. M., R. Hartman, K. Alstad, and C. Pien. 2023. zooper: an R package to download and integrate zooplankton datasets from the Upper San Francisco Estuary. Zenodo. doi:10.5281/zenodo.3776867

CBP 2023. Chesapeake Bay Program. <https://www.chesapeakebay.net/>

CDFW a. 2023. California Department of Fish and Wildlife San Francisco Bay study: Long-term fish and water quality monitoring data. <https://filelib.wildlife.ca.gov/Public/BayStudy/>

CDFW b. 2023. Interagency Ecological Monitoring Program. https://iep.ca.gov/Science-Synthesis-Service/Monitoring-Programs/EMP

Clark J, Bashevkin SM. 2022. deltafish: an R package to access an integrated dataset of fish counts and lengths from the San Francisco Estuary v0.1.0. Zenodo. doi:10.5281/zenodo.6484440

Comte, L., Grantham, T., & Ruhi, A. (2021). Human stabilization of river flows is linked with fish invasions across the USA. Global Ecology and Biogeography, 30(3), 725-737.

Hampton, S.E., Holmes, E.E., Scheef, L.P., Scheuerell, M.D., Katz, S.L., Pendleton, D.E. and Ward, E.J., 2013. Quantifying effects of abiotic and biotic drivers on community dynamics with multivariate autoregressive (MAR) models. Ecology, 94(12), pp.2663-2669.

Latour, R. J., Gartland, J., & Bonzek, C. F. (2023). Design and redesign of a bottom trawl survey in Chesapeake Bay, USA. *Frontiers in Marine Science*, *10*.

Lojkovic Burris, Z. P., Baxter, R. D., & Burdi, C. E. (2022). Larval and juvenile Longfin Smelt diets as a function of fish size and prey density in the San Francisco Estuary. California Fish and Wildlife Journal, 108, e11.

MWRA 2023. Massachusetts Water Resources Authority Water Column Monitoring Program. https://www.mwra.com/

R Core Team (2023). R: A language and environment for statistical computing. R Foundation for Statistical Computing, Vienna, Austria. <https://www.R-project.org/>

Tu, T., Comte, L. and Ruhi, A., 2023. The color of environmental noise in river networks. Nature Communications, 14(1), p.1728.

Viechtbauer, W (2010). “Conducting meta-analyses in R with the metafor package.” Journal of Statistical Software, 36(3), 1–48. doi:10.18637/jss.v03
